# A review on the green and sustainable synthesis of silver nanoparticles and one-dimensional silver nanostructures

**DOI:** 10.3762/bjnano.12.9

**Published:** 2021-01-25

**Authors:** Sina Kaabipour, Shohreh Hemmati

**Affiliations:** 1 School of Chemical Engineering, Oklahoma State University, Stillwater, Oklahoma, 74078, USA

**Keywords:** green nanotechnology, green reagents, green synthesis, one-dimensional silver nanostructure, silver nanostructures

## Abstract

The significance of silver nanostructures has been growing considerably, thanks to their ubiquitous presence in numerous applications, including but not limited to renewable energy, electronics, biosensors, wastewater treatment, medicine, and clinical equipment. The properties of silver nanostructures, such as size, size distribution, and morphology, are strongly dependent on synthesis process conditions such as the process type, equipment type, reagent type, precursor concentration, temperature, process duration, and pH. Physical and chemical methods have been among the most common methods to synthesize silver nanostructures; however, they possess substantial disadvantages and short-comings, especially compared to green synthesis methods. On the contrary, the number of green synthesis techniques has been increasing during the last decade and they have emerged as alternative routes towards facile and effective synthesis of silver nanostructures with different morphologies. In this review, we have initially outlined the most common and popular chemical and physical methodologies and reviewed their advantages and disadvantages. Green synthesis methodologies are then discussed in detail and their advantages over chemical and physical methods have been noted. Recent studies are then reviewed in detail and the effects of essential reaction parameters, such as temperature, pH, precursor, and reagent concentration, on silver nanostructure size and morphology are discussed. Also, green synthesis techniques used for the synthesis of one-dimensional (1D) silver nanostructures have been reviewed, and the potential of alternative green reagents for their synthesis has been discussed. Furthermore, current challenges regarding the green synthesis of 1D silver nanostructures and future direction are outlined. To sum up, we aim to show the real potential of green nanotechnology towards the synthesis of silver nanostructures with various morphologies (especially 1D ones) and the possibility of altering current techniques towards more environmentally friendly, more energy-efficient, less hazardous, simpler, and cheaper procedures.

## Review

### 1 Introduction

Nanotechnology has been ubiquitously applied in almost every scientific discipline. Nanomaterials have been utilized in innumerable applications due to their unique characteristics. Novel, successful applications of nanomaterials and nanostructures can be seen in drug delivery [1–6], nanomedicine [7–10], food packaging [11–13], aseptic procedures [14–16], correlative microscopy [17], imaging [18–22], optics [23–24], microelectronics [25–27], three dimensional (3D) printing [27–31], renewable energy [32–36], wastewater remediation [37–38], and catalysis [39–43], to name a few. The success of nanotechnology has been established and the promising outcomes cannot be overlooked; however, the main principles behind the production of nanomaterials are yet to be examined more closely in terms of economy as well as effects on health and environment.

Among metal nanostructures, silver nanostructures have demonstrated promising potential in many applications and have contributed significantly to the advancement of nanoscience. The concept of using silver is not unprecedented; silver was used broadly by many nations and dynasties throughout ancient history. The early applications of silver date back to 4000 B.C.E, by the Caldeans [44], and throughout ancient history, Persians, Romans, Egyptians, and Greeks utilized silver for food storage purposes [45]. Silver was also extensively used in utensils for eating and drinking, most probably due to the antimicrobial activities of silver which were discovered from experience during ancient centuries [45]. Later on, there were further instances where silver was used for medical purposes. For instance, Avi-Cenna applied silver filings for blood purification and to inhibit heart palpitations [44]. Silver was used in forms of aqueous solutions, coins, and plates for aseptic and antibacterial applications, as well as in food and dairy preservation up until the 19th century, as it was agreed that food and dairy products kept in silver containers lasted longer than those in other types of containers [44]. Since then, silver has been and is still in use in accordance with these old traditions even today. Although silver has proved its potential and advantages, the mechanisms behind its functionality were not well understood in the past. Nanotechnology has emerged as a means to delve further into the usefulness of this precious element.

Silver nanostructures can be categorized based on their shape and morphology. Different shapes and morphologies of silver nanostructures have been synthesized, including cubes [46–47], spheres [48–50], triangles [51–53], prisms [54–55], sheets [56–58], disks [59–60], rods [61–62], bars [63–64], and wires [65–68].

Silver nanostructures have received significant attention in the last decades due to their improved properties compared to bulk silver and their unique intrinsic characteristics including antimicrobial activity, electrical conductivity, thermal conductivity, optical characteristics, and mechanical properties. The antimicrobial characteristic of silver nanoparticles (AgNPs) has made them highly applicable in the biomedical and therapeutic fields [69–71]. Currently, antimicrobial AgNPs can potentially act as alternatives for current antibiotics due to increased bacterial resistance [71]. In addition, the development of new generations of antibiotics is costly, which prevents pharmaceutical companies from manufacturing new classes of antibiotics [72–73]. Furthermore, the physicochemical characteristics of AgNPs can be tuned in a way to avoid cellular toxicity [71,74–75], which facilitates their biomedical applications. The small size of AgNPs (<100 nm) allows them to accumulate on the extracellular membrane of the bacteria and penetrate inside, which alters the membrane permeability and leads to bacterial death [71,76]. Another therapeutic approach lies in the bactericidal activity of functionalized silver nanoparticles coated on surfaces. This method is applicable in developing aseptic catheters to prevent catheter-related infections such as urinary tract and venous infections, and also inhibit the growth of bacterial biofilms [14–16].

AgNPs were also used in developing strong thermally conductive materials. They were used in polymer composites to increase thermal conductivity (K) [77–78] for cooling applications in electronic equipment. Furthermore, AgNPs have demonstrated unique electrical properties. AgNPs coated on polycarbonate substrates were previously used to increase the electrical conductivity of polycarbonate composites [79]. AgNPs have also demonstrated minimum or no adverse effects on mechanical strength when embedded in polymeric materials or composites [80–82]. For instance, the utilization of AgNPs in bone cement is meant to prevent bacterial infection while sustaining the mechanical strength of the cement connected to the prosthesis [82]. AgNPs have also demonstrated significant optical properties. They possess substantial surface plasmon resonance (SPR) and generally have a broad absorption spectrum [83]. This enables applications in optoelectronics and surface-enhanced Raman scattering [84–85]. AgNPs were also applied effectively in solar cell matrices [32,86–87]. AgNPs can enhance the current density in solar cells due to their far-field effect and localized surface plasmon resonance (LSPR) [32].

There are several applications in which use of 1D silver nanostructures such as nanowires (NWs) and nanorods (NRs) (at the same concentration) are preferred to other nanostructures due to stronger conductivity. For instance, 1D silver nanostructures can provide desired electrical characteristics in conductive adhesives at lower concentrations compared to other silver nanostructures and micrometer-sized ones [88]. The research being conducted on the synthesis of silver nanowires (AgNWs) is currently gaining a lot of attention due to their promising applications in electronics. This is a result of the outstanding electrical, optical, and mechanical properties of AgNWs. 1D silver nanostructures, such as AgNWs are more advantageous compared to other silver nanostructures due to several reasons. They can enable free movement of electrons in one direction [89], and can form networks of wires that facilitate the passage of electrical current. They can also improve transmittance characteristics due to their high aspect ratio [90]. Novel applications can be seen in transparent conductive films (TCFs) [91], wireless technology [92–93], touchscreen devices [94], organic light emitting diodes (OLED) [95], transparent conductive electrodes [96–97], artificial skin [98], liquid crystal display (LCD) [99–100], and smart windows [101–102]. AgNWs can be embedded in flexible touch-screen substrates and electronic displays to provide an enhanced decrease in sheet resistance and to increase touch sensitivity [89]. Furthermore, AgNWs can be used to prepare AgNW-based conductive inks that have remarkable rheological characteristics such as thixotropic shear thinning and thus, can be simply used for screen printing without the addition of polymeric rheological additives [103]. AgNW-coated conductive films have been considered as a promising alternative over conventional indium tin oxide (ITO)-coated conductive films [89].

The synthesis of silver nanostructures, and generally all types of nanostructures, can be categorized as one of two approaches [104]. The first one is the top-down approach where particles are produced from the bulk material, and the second one is the bottom-up approach where nucleation sites are formed and finally grow into a nanometer-sized particle. The first approach consists of a set of techniques also known as “physical” synthesis methods. Several physical methods for top-down synthesis of silver nanostructures including ball milling [105–107], laser ablation [108–112], evaporation–condensation [113–114], electromagnetic levitation gas condensation (ELGC) [115], ultrasonication [116–119], lithography [120–121], spray pyrolysis [122–124], radiolysis [125–128], arc discharge [129–133], and photoirradiation [134–136] have been utilized to synthesize various morphologies of silver nanostructures with varied size and size distribution. The physical synthesis method is primarily used for large-scale production in a short amount of time [137].

The bottom-up approach, however, mostly relies on the use of reducing agents for the production of silver nanoparticles. This approach is also categorized into two distinguishable, but not completely disparate, set of methods. The first category consists of techniques that use chemical reagents to reduce Ag cations into zero-charged Ag atoms, which then mount on top of the nuclei, serving as templates for crystal growth into particles at the nanoscale [138–139]. This set of techniques is also known as the “chemical” synthesis method. These techniques are usually accompanied by the addition of stabilizers to provide stability, prevent aggregation, control morphology, and provide physiologically compatible properties [140–142]. Chemical methods were previously used to produce silver nanoparticles including sol–gel processes [143–146], conventional chemical reduction [147–151], reverse micelle [152–154], co-precipitation [155], chemical vapor deposition [156–158], solvothermal [159–161], and electrochemical reduction [162–165]. Chemical synthesis methods are currently among the most widely used approaches [104,166]. The second category in bottom-up synthesis methods consists of a set of techniques that incorporate the use of non-chemical reagents for the synthesis of silver nanostructures. Those techniques rely on the use of biological agents or bio-extracted compounds. The term used for addressing these methods is also referred to as “biological” synthesis. Previous studies have used bacteria [167–170], fungi [171–174], viruses [175–176], yeasts [177–179], plants [180–183] and plant extracts [166,184–188], microalgae [189–193], enzymes [194–196], saccharides [197–201], and vitamins [202–204] to synthesize Ag nanostructures with controlled size and morphology. These methods can also be referred to as a subcategory of green synthesis.

Numerous works in the literature have focused on synthesis of AgNPs without giving much attention to the disadvantages such as cost, time, and hazards. Nevertheless, other essential factors such as cost-effectiveness, eco-friendliness, energy consumption, and human well-being can easily be overlooked. For instance, hydrazine, as a strong and common reductant [205–207] for the synthesis of AgNPs [149,208–211] is a highly toxic, cancerous, and lethal chemical [205]. Although some works have incorporated green synthesis techniques [111–112,135,164,212–213], they do not completely eliminate the need for chemicals, nor do they rule out high-energy consumption in physical synthesis methods, thereby increasing costs greatly [137,214].

Due to the disadvantages of physical and chemical methods, the focus has been directed towards the use of facile methods and materials that are less detrimental and more cost-effective. A common subject of interest in this area has been the selection of naturally occurring processes, widely known as “green processes”. However, green synthesis of silver nanostructures is a general term defined as the production of silver nanostructures by using environmentally friendly techniques. Therefore, this method is not limited to synthesis by biological agents. For instance, alternative reagents such as ascorbic acid and sodium citrate are considered to synthesize AgNPs in a green and environmentally friendly manner [215]. The green synthesis of silver nanostructures has been receiving significant attention and is expected to rule out the use of hazardous chemical substances to a great extent [45,216–218], and also to disregard the use of high-energy consuming devices for the top-down synthesis of silver nanostructures [104,185]. Green synthesis of silver nanostructures has demonstrated great advances in the last decade, yet there are still issues regarding stability, size distribution, morphology, unknown biological functions [140], and the consideration that some biological processes cannot become industrially feasible due to strict and time-consuming aseptic procedures [185].

In this review, we first aim to discuss the most common AgNP synthesis methodologies and to compare them based on their cost, eco-friendliness, and energy consumption to show how green chemistry can improve the process and act as an alternative compared to physical and chemical synthesis. The physical and chemical synthesis methods are discussed only in terms of the process structure, advantages, and disadvantages. Therefore, a detailed study of physical and chemical methods for the synthesis of AgNPs is out of the scope for this review. Among the green synthesis procedures, significant attention has been recently given to plant-mediated synthesis because of its simple culturing procedures and potential for scale-up [181,219]. Various green synthesis studies from the literature were gathered and compared, giving one a clear and broad overview of the green synthesis processes. We then show how plant-mediated synthesis can emerge as a novel and alternative methodology towards the synthesis of 1D silver nanostructures, which have several applications in electronics. Finally, novel future directions including application of in situ characterization techniques in the course of reaction, continuous green and sustainable synthesis of silver nanostructures adaptable for in situ characterization, and incorporation of artificial intelligence (AI) in green silver nanostructures synthesis are discussed.

### 2 Physical and chemical synthesis methodologies of silver nanoparticles

In this section, chemical and physical synthesis methodologies of silver nanostructures are reviewed and their advantages and disadvantages are discussed. This section is reviewed to provide a comparison between chemical/physical synthesis and green synthesis of silver nanostructures. Since the main focus of this article is on reviewing the green synthesis of silver nanostructures as an alternative over chemical and physical methods, only the most common physical and chemical methods have been reviewed and the major part of our review has been dedicated to green synthesis of silver nanostructures.

#### 2.1 Physical synthesis

Physical synthesis of silver nanoparticles includes methods that produce particles with dimensions of 1–100 nm from bulk silver, typically in the solid phase. It is also referred to as the top-down synthesis of silver nanoparticles. Unlike chemical and biological methods, physical techniques do not necessarily require using a reducing agent or stabilizer; however, they may be incorporated with other techniques.

**2.1.1 Ball milling process.** As one of the conventional processes, the ball milling process (mostly used as mechanochemical ball milling) is a method that is used commonly to produce AgNPs in a solid state [220]. Previously, AgNPs were produced using high-energy planetary ball milling [105–106,221]. Khayati et al. [105] utilized planetary ball milling in a mechanochemical process by adding organic process control agents (PCAs). In this work, depending on the type of PCA used, particle sizes varied from 14 to 34 nm, and were of crystallite shape. In another work, AgNP crystallites were produced with an average size of 10–12 nm using the mechanochemical ball milling process by utilizing polyethylene glycol as the stabilizing agent [107]. The relatively small size of the produced AgNPs allowed effective antibacterial activity against Gram-positive and Gram-negative bacteria. The ball milling method is an inexpensive approach for the synthesis of AgNPs in a solid state [140], and can be used for the synthesis of AgNPs in ambient temperature, with a fair control over particle size [107]. It is a useful technique and synthesized nanoparticles may be used for antimicrobial applications [107]; however, there are several downsides with this method. The most common approach, provided by the literature, for the synthesis of NPs – especially AgNPs – is by laboratory-based planetary ball milling, which is insignificant for large-scale production [222]. In addition, the milling process itself may result in the creation of agglomerated products, especially during long processes, due to the large specific surface area of the produced NPs [222–223]. Furthermore, this technique is associated with substantial energy consumption – considering the milling period – compared to alternative methods [222].

**2.1.2 Evaporation–condensation process.** Evaporation–condensation is another conventional method used for the synthesis of AgNPs. This technique is applied by using a furnace processing chamber where the metal of interest is vaporized into a low-density gas phase, becomes supersaturated by decreasing temperature, and then is condensed to form nuclei which then grow into nanoparticles [113,137]. The chamber gas usually contains an inert gas such as helium or argon [113,224]. AgNPs synthesized using this technique were of crystalline shape with sizes from 7 to 55 nm [113,224]. One of the advantages of this method is that it can potentially produce NPs on a large-scale [137], and can be used for long-term experiments [104,225]; however, there are significant disadvantages to using this technique. The required equipment is rigorous, very costly, and occupies a significantly large space [104,137,214]. Furthermore, due to the high operation temperature, a significant preheating time is required, and the process needs considerable time to achieve thermal stability [214,225]. There are also safety issues due to the high processing temperature which will elevate the surrounding environment temperature [137,226]. Additionally, the process consumes a significant amount of energy [214,225] due to the very high operating temperatures; thus, making it uneconomical.

**2.1.3 Arc discharge process.** Another physical method widely used for the synthesis of AgNPs is the arc discharge method. In this method, two electrodes – a cathode and an anode – are connected in a high current DC circuit and submerged in a solvent – mostly deionized water – to run the process [129,131]. These electrodes can be either composed of an inert metal, such as titanium, or any metal of interest of which the nanoparticles will be produced, for instance, silver for the synthesis of AgNPs [129,131]. In the case of titanium electrodes, AgNO_3_ is used as the precursor, an electric discharge takes place between cathode and anode, and an electron exchange takes place in the plasma region where silver ions are reduced [129]. In the case of silver electrodes, silver will be melted and vaporized from the electrode ends, and as a result, nanoparticles are formed from the silver condensates [131]. Tien et al. [227] synthesized AgNPs from 5 to 45 nm using the same method. In more recent works by Tseng et al. [132–133], AgNPs were produced using a micro-electrical discharge machining system and by adding polyvinyl alcohol (PVA) as the capping agent. They obtained AgNPs with a diameter of 50–100 nm when PVA was not used, and a diameter in the range of 25–75 nm when PVA was used [132]. The arc discharge method is advantageous in terms of the simplicity of the apparatus and equipment, low impurity due to the mere use of water, and fewer production steps [129]. In addition, this process can reach high NP synthesis rates in a short time [129,131]. However, the size distribution is large [129,132–133], and the NPs produced have a fairly large size distribution compared to NPs produced by chemical methods.

**2.1.4 Laser ablation process.** A promising physical synthesis method used widely in recent years is laser ablation. This method is typically used for the synthesis of stable silver colloids either in solutions or in open air without utilizing any additional reagents [108,112]. AgNPs synthesized with this method are maintained in high purity due to the absence of chemical stabilizers and ligands, which provide NPs with unique surface characteristics [228]. Therefore, for safe biological applications (i.e. in the medical and food industry), this approach would be preferred as an alternative for methods that necessarily require the use of chemical stabilizers [108,112]. In addition, the small NP size, low agglomeration rate, and narrow size distribution can be achieved by this method [108]. The process uses a laser beam at high energy to ablate pure Ag from which the separated AgNPs, either in liquid or vapor form, are attained and confined in the surrounding ambient [109]. The formation of nanoparticles by laser ablation depends on the thermal and optical properties of the utilized metal and the surrounding ambient conditions [228–229]. Despite its significant advantages, laser ablation presents some disadvantages that limit their use. In general, this method does not have high productivity and the utilization of laser ablation at an industrial scale is difficult. To achieve desired concentrations, high-energy lasers should be used, which increase the costs significantly [112].

**2.1.5 Spray pyrolysis process.** Spray pyrolysis is another method used to produce AgNPs with an average size of 10 nm embedded into amorphous calcium phosphate particles for enhanced adhesive applications [124]. The spray pyrolysis process requires using an atomizer, a tube furnace, a reaction tube, a collection filter, and a vacuum pump [124]. This method is also often used for production of metal powders and demonstrates less agglomeration, higher purity, and higher crystallinity compared to those produced by chemical methods [230]. The method is simple and reproducible [230]; however, the process runs using high operating temperatures, and more specifically, the center of the reaction tube may not reach the setpoint temperature due to the short residence time inside the reactor and finite heat transfer from the wall [124].

In general, although physical methods can produce nanoparticles with high purity, most of them are very expensive and may lead to agglomeration of products [140]. Based on all the disadvantages explained here, the mere use of physical methods may not be adequate for most cases to produce AgNPs with desired size, morphology, size distribution, and characteristics. Moreover, most physical approaches should be accompanied with chemical or green methodologies to compensate for deficiencies.

#### 2.2 Chemical synthesis

Chemical reduction, or conventional chemical synthesis, is the most common approach for the synthesis of AgNPs [214]. This is performed by the presence of a metal precursor such as AgNO_3_, a reducing agent such as hydrazine, sodium borohydride, ethylene glycol, or dimethylformamide (DMF) as well as the presence of a stabilizer such as polyvinylpyrrolidone (PVP) or polyvinylalcohol (PVA). The chemical synthesis of nanoparticles is one of the bottom-up techniques due to the fact that particles are formed from collective atoms in a nucleus rather than from the bulk [104].

**2.2.1 Sol–gel process.** The sol–gel method is one of the most common techniques to synthesize AgNPs. The sol–gel process is considered to be a multifaceted approach for the synthesis of nanoparticles in various forms – especially complex compounds – such as metal-complex oxides, inorganic nanocomposites, and chalcogenides [144]. In the sol–gel method, a gel-like mixture is first prepared by mixing the silver precursor solution with a metal complex compound (i.e. comprised of Ca, Ti, Sr, etc.) [143–144] in a solvent such as water or alcohol. Then the product is heated in order for the nucleation and reaction to take place [143,231]. In most cases, AgNPs are synthesized in metal oxide thin films such as TiO_2_, SiO_2_, and ZrO_2_ where the average particle size is almost 10 nm when the heating temperature is 600 °C in the case of SiO_2_ thin films, and 500 °C in the case of TiO_2_ and ZrO_2_ [231]. Arun Kumar et al. [145] synthesized AgNPs using the hydrolytic sol–gel method at 400, 600, and 800 °C with particles with an average size of 20 nm and crystalline shape. The sol–gel technique can also be performed at lower temperatures as well. Jadalannagari et al. [143] synthesized silver-doped hydroxyapatite nanorods using the sol–gel technique at 100 °C and produced particles with an average diameter of 25 nm with hexagonal cross section. In the sol–gel process, besides temperature and gel composition, the solvent plays an important role in determining the size, morphology, and surface characteristics of the synthesized AgNPs [232]. Organic solvents are generally more advantageous, as they can act as an oxygen-supplying agent for the metal oxide and result in more uniform structures and a smaller size distribution [232]. One important advantage of the sol–gel technique is the large freedom for choosing different precursors with various combinations (i.e. hybrid compounds), allowing the process to be adjusted accordingly in order to yield the desired complex product with tuned physiochemical characteristics [146]. In addition, combined with the hydrothermal approach (synthesis in a hot aqueous environment under high pressure), it can synthesize AgNPs at a lower temperature compared to the sol–gel process alone [144]. Nevertheless, there are some disadvantages regarding the application and feasibility of sol–gel-produced nanoparticles and nanocomposites. For instance, in industrial applications of nanoparticle-doped glasses such as those in the automotive industry, there exists some difficulty in the production of thick films larger than 1 µm because of possible film cracks and shrinkage [233]. In addition to that, the film quality itself depends highly on process and environmental conditions such as temperature and humidity [233]. The sol–gel process is associated with costly precursors, process longevity, and difficulties regarding reproducibility [234].

**2.2.2 Reverse-micelle process.** The reverse micelle is another approach for the synthesis of AgNPs. Reverse micelles are produced from surfactants such as sucrose fatty acids in a hydrophobic solvent such as alkanes [235]. There is a water phase inside the microemulsions which is also referred to as the water pool where the reactants are present [235]. The water pool is where the silver ions are reduced into silver atoms which then form AgNPs [152]. The reverse micelle method has served as a common approach for the synthesis of AgNPs throughout the past two decades [152]. Regular reducing agents used in this method include sodium borohydride (NaBH_4_) [236], hydrazine (N_2_H_4_) [237], glucose [238], and quercetin [152,239], to name a few. The size and size distribution of the AgNPs is controlled by the strength of the reducing agent [152]. It was previously observed that hydrazine hydrate (N_2_H_4_·H_2_O) can yield smaller AgNPs with a higher degree of dispersion compared to stronger reductants such as sodium borohydride (NaBH_4_) [240]. Singha et al. [152] synthesized AgNPs in sodium dioctyl sulfosuccinate (AOT) reverse micelles using ascorbic acid as the reductant and obtained particles with an average size of 6 nm. Yang et al. [154] used sodium borohydride as the reductant and octadecylamine (ODA) as the solvent and produced AgNPs with an average size of 3.38 nm. The size distribution of the synthesized nanoparticles depends on the type of solvent as well as the reducing agent. This enables a method that provides many choices depending on the type of surfactant and solvent, which can be optimized to yield the desired size and morphology [152]. In addition, preparation of AgNPs using this method does not need any specific equipment, intense temperature, or pressure conditions [235]. The scale-up of the system is also relatively simple [235]. However, the downside of this method is the low productivity of particles per volume as a result of low reactant concentration inside the reverse micelles [235]. AOT-microemulsions have been the most common microemulsions for the preparation of micelles [152]. However, this method may result in the synthesis of AgNPs with weak surface plasmon characteristics due to a broad surface plasmon band [152].

**2.2.3 Chemical vapor deposition process.** Chemical vapor deposition (CVD) and atomic layer deposition (ALD) are among other chemical methods for nanoparticle synthesis. CVD is a method that allows production of nanoparticles on a substrate [241]. The process consists of three steps. First, the addition of a volatile precursor in the gas phase to the reactor chamber. Second, adsorption of the vapor on the substrate surface and establishment of medium compounds followed by formation of a layer. Third, nucleation and growth of the layer through heating [241]. The important factors that control the process and size of synthesized AgNPs include the precursor introduction method, reactor pressure, gas flow properties, deposition rate, deposition duration, and substrate surface temperature [157,241]. The type of precursor appears to be the most significant factor in the process [241]. Silver nitrate is the most widely used precursor for this purpose [241]. An important advantage of this technique is the establishment of a silver-metal-oxide (e.g. SiO_2_ or TiO_2_) nanocomposite coating using only one deposition step [241]. The CVD method also provides many opportunities for the synthesis of silver-coated materials with varying size distribution and morphology [241]. The disadvantages of this method are associated with the high process cost, complexity, and low scale-up capability [158].

**2.2.4 Wet chemical synthesis.** Currently, most synthesis methods still rely on wet chemical reduction using a chemical reducing agent. The conventional wet synthesis of AgNPs using strong reductants such as sodium borohydride, hydrazine, and dimethylformamide (DMF) is currently the most common approach in the literature compared to other techniques previously described [214]. Although wet chemical methods can successfully offer narrow size distribution and can synthesize particles with small size, there are some major disadvantages regarding these methods. Additionally, toxicity and hazards can result from using these chemical substances. For instance, hydrazine and its derivative compounds (e.g. hydrazine hydrate) serve as a strong reductant in the synthesis of AgNPs [211] due to its strong reducing characteristic [242], however; hydrazine is known to be toxic and carcinogenic and causes severe damage to vital human organs such as the lungs [242–243]. The Environmental Protection Agency (EPA) has ranked hydrazine as a potential carcinogen with threshold limits as small as 10 ppb [242,244]. *N*,*N*-Dimethylformamide (DMF) is also known as a strong and common reducing agent. However, the compound is reported to cause damage to the liver and digestive system [245]. Sodium borohydride, another strong reducing agent, is considered to have adverse effects on lungs and may cause pulmonary edema, which is a condition that results in fluid build-up in the lungs [246]. Besides the exposure risk during the process, an intense separation step should be considered to remove those compounds from the synthesized nanoparticles, thus making the process difficult and costly. In the case of hydrazine, the synthesized particles may potentially include some of the remnants from the reagent, thus making them hazardous or even unusable for biomedical applications. The polyol process is another common wet chemical method used for the synthesis of silver nanostructures. The polyol process is typically performed at 120–160 °C by utilizing ethylene glycol as the solvent and reducing agent and PVP as the capping agent in the presence of a small amount of salt mediator [247–249]. Although the polyol process is a non-hazardous and widely accepted method for the synthesis of silver nanostructures, there are some disadvantages associated with it. First, the polyol process is typically performed at temperatures higher than 120 °C, which is associated with high energy consumption. Second, this process requires dilute concentrations of silver precursors (0.1 M or lower) in order to maintain a uniform morphology and size distribution [250], which limits their scale-up capability. Third, the yield of the silver nanostructures is highly sensitive to reaction conditions, such as temperature, concentration ratio of PVP to AgNO_3_, salt mediator concentration, and the stirring type and rate [90]. On the other hand, natural compounds can be utilized to synthesize silver nanostructures in a much simpler fashion by enabling synthesis at room temperature and using only one reagent as both reducing and capping agent in addition to providing a non-hazardous and environmentally friendly route towards the synthesis of different silver nanostructures.

### 3 Green synthesis of silver nanoparticles

**Figure 1:**
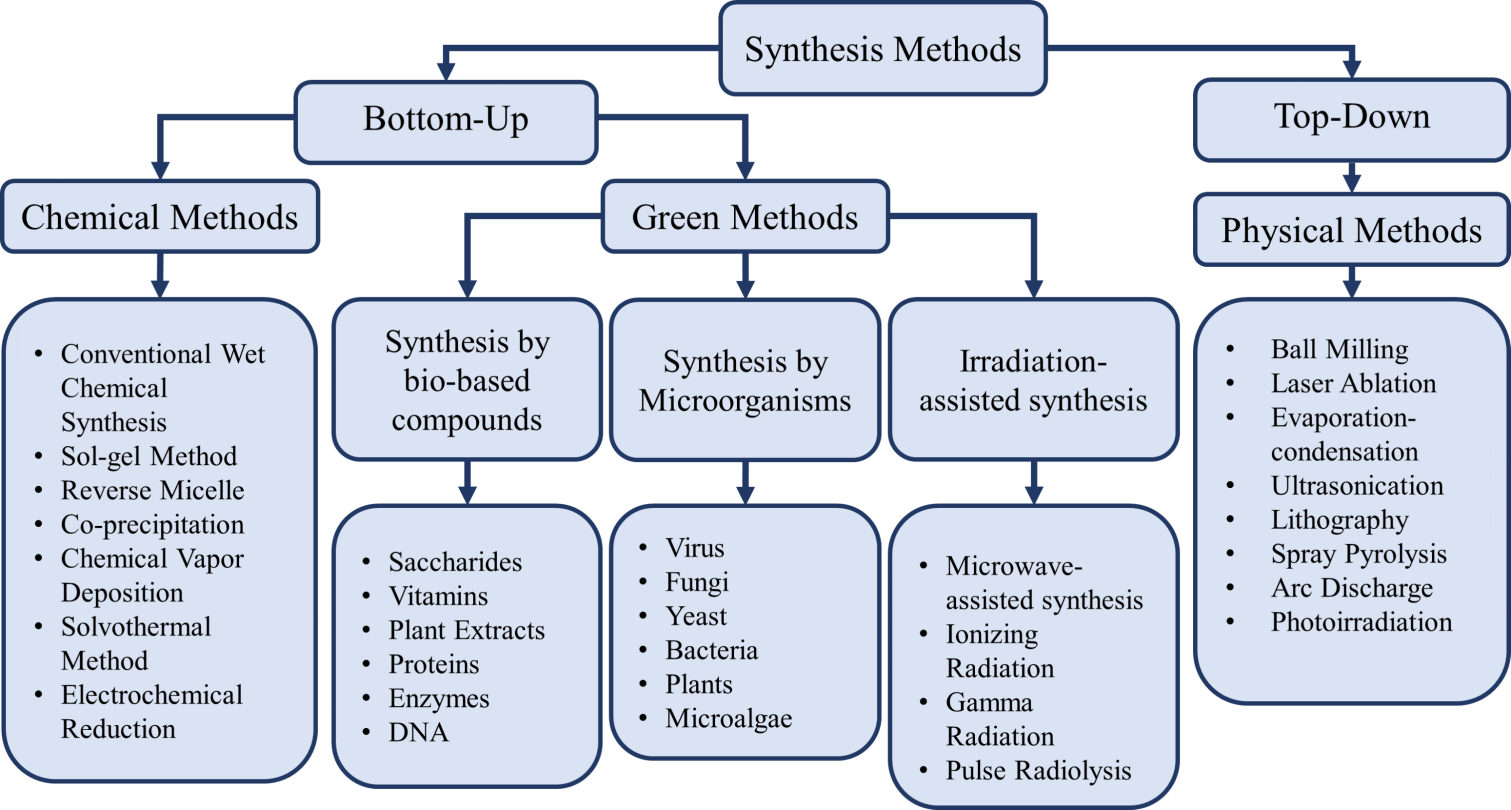
Classification of physical, chemical, and green synthesis methodologies of silver nanostructures with respect to reducing agent type, instruments, and processes.

The green synthesis of nanoparticles can be defined as a set of techniques which utilize non-chemical reagents or non-hazardous methods for the production of nanoparticles. The main purpose of such methodologies is to minimize environmental toxicity and health-related hazards [104,214,251–253]. In most green silver nanostructure synthesis processes, the reduction of Ag^+^ to Ag^0^ is performed either by biological species or by bio-based compounds derived from a desired type of plant or organism [252], which is also called biological synthesis. Bio-based reducing agents include microorganisms or biologically produced material. Microorganisms consisting of viruses, microalgae, fungi, yeast, and bacteria were previously used to synthesize AgNPs. Unlike physical synthesis, but similar to chemical synthesis, the biological synthesis of nanoparticles is classified under bottom-up approaches [104]. However, green synthesis is not limited to synthesis by bio-based compounds or biological species. For example, laser irradiation can also be used without using any reducing agent in order to synthesize AgNPs [252]. In addition, AgNPs were synthesized by microwave irradiation [254–255], ionizing irradiation [252,256], and pulse radiolysis [252]. Since these techniques may be utilized to produce nanoparticles with harmless procedures, they may also be classified under green synthesis methodologies. However, they have drawbacks in terms of energy consumption. A general comparison between chemical, physical, and green synthesis methods has been outlined in Table 1. Figure 1 provides a clear overview of the classification of synthesis methods.

**Table 1:** Classification and comparison of various synthesis methodologies of silver nanostructures based on their advantages and disadvantages as reported in the literature.

Method	Advantages	Disadvantages	Ref.

Physical methods			

ball milling	• cheap • process can be done at ambient temperature	• insignificant for large-scale synthesis • agglomeration of particles • high energy consumption	[140,222–223,257–258]

evaporation–condensation	• large-scale synthesis • useful for long-term experiments	• rigorous equipment • expensive equipment • expensive process • long preheating times • high operating temperatures • high energy consumption	[104,137,214,225]

arc discharge	• simple equipment • high purity of nanoparticles • few process steps • high synthesis rates	• large size distribution of nanoparticles	[129,131–133]

laser ablation	• high purity of nanoparticles • small size of nanoparticles • low agglomeration rate • narrow size distribution	• high energy consumption • low productivity • insignificant for large-scale synthesis	[112,228–229]

spray pyrolysis	• high purity of nanoparticles • process simplicity	• high operating temperatures • low heat transfer to the reaction tube due to low residence time	[124,140,230]

Chemical methods			

sol–gel process	• high purity and homogeneity of nanoparticles • can be efficiently used for preparation of composites and complex materials	• limited industrial applicability • costly precursors • process longevity • difficulties regarding the synthesis of monoliths • reproducibility difficulties	[233–234]

reverse micelle	• process simplicity • scale-up simplicity	• low productivity of particles per system volume • large size distribution of nanoparticles • use of toxic and hazardous chemicals such as hydrazine and sodium borohydride	[152,235]

chemical vapor deposition	• preparation of nanocomposites by only one deposition step	• high process cost • process complexity • weak scale-up capability	[158,241]

wet chemical synthesis	• process simplicity • narrow size distribution of nanoparticles • small size of nanoparticles	• use of highly toxic and hazardous substances such as hydrazine, sodium borohydride, and *N*,*N*-dimethylformamide (DMF) • limited biomedical applications	[242–245]

Green/biological methods			

bacteriogenic synthesis	• process simplicity • environmentally-friendliness	• pathogenic behavior of certain species such as *E. coli* • significantly slow synthesis rate • large size distribution • unknown biological functions affecting the synthesis process	[104,259]

fungi-mediated synthesis	• process simplicity • environmentally-friendliness • faster synthesis rate compared to bacteriogenic synthesis • high bioaccumulation capacity and intracellular uptake • less non-pathogenic behavior compared to bacteriogenic synthesis	• process longevity • pathogenic behavior • unknown biological functions affecting the synthesis process	[104,178–179,260]

virus/VLP-mediated synthesis	• process simplicity • environmentally-friendliness • possibility to synthesize 1D nanostructures • small size of nanoparticles	• lack of strong metal-binding sites along the biotemplate surface • preparation of the biotemplate is time-consuming • multiple coating cycles may be required to yield a uniform coating	[261–263]

algae-mediated synthesis	• process simplicity • low cost • environmentally-friendliness • low reaction temperatures • use of non-pathogenic and non-hazardous reagents • small size of nanoparticles • uniform morphology of nanoparticles	• significantly Slow synthesis rate • unknown biological functions affecting the synthesis process	[264–265]

plant/plant extract-mediated synthesis	• process simplicity • low cost • environmentally-friendliness • low reaction temperatures • use of non-pathogenic and non-hazardous reagents • they can act as both reducing and capping agent at the same time • broad scope	• unknown mechanisms affecting the synthesis process	[104,140,187–188]

Nature has proven its unique ability to provide advice for synthesizing nanomaterials. The biogenic/green metal nanostructures’ future is bright. Green processes are highly advantageous since they utilize naturally occurring processes to synthesize nanoparticles. The idea was first introduced in the 19th century when scientists discovered the reducing ability of biological materials [130]. Green/biological methods provide several advantages over physical and chemical methods. They are eco-friendly unlike chemical methods [45,104], require less energy unlike physical methods [104,214], can be used for mass production [181], and are more economically feasible [104,181,219]. Sustainability, energy efficiency, renewability, and reduction of chemical derivatives, are among other advantages of green synthesis processes [266]. Furthermore, nature provides a high variety of reagents that can act as reducing agents, while in chemical methods the reagent selection including reducing, stabilizing, and capping agents are more limited. Therefore, recent works in the literature introduce highly diverse biological and green methods for the synthesis of AgNPs. However, it is critical to optimize these techniques not only in terms of scale-up capability, but also with respect to product quality and performance. Various green synthesis studies of Ag nanostructures are reported in Table 2 along with the reaction conditions, size and morphology of synthesized Ag nanostructures, responsible functional groups in reduction, nucleation and growth, and applications of the synthesized Ag nanostructures.

**Table 2:** A review of various green processes, reaction conditions, necessary functional groups, and characterization techniques used for the synthesis of silver nanostructures with different sizes and morphologies along with their application.

Reducing agent	Solvent	Ingredient or functional group responsible for Ag^+^ reduction/particle stabilization	Stabilizer	Reaction time/ temperature
	
	Morphology/ dimensions	Implemented characterization techniques	Application of the synthesized Ag nanostructures	Ref.

Bacteria/ bacteriogenic metabolites				

exo-polysaccharide (EPS) extract of *Bacillus safensis* LAU 13	not explicitly mentioned	secondary amine and amide (responsible for stabilization)	N/A	2 h/30 ± 2 °C
	spherical/5–30 nm	FTIR/TEM/XRD	antimicrobial agents	[269]

*Bacillus krulwichiae*	distilled water	carboxyl, hydroxy, aldehyde, ester groups responsible for reduction and stabilization	N/A	24 h/room temperature
	spherical/av.: 25.88 ± 10.49 nm	FTIR/SEM/XRD/EDX	antimicrobial/anti-biofilm agents	[280]

*Bacillus cellulosilyticus* (EPS extract)	distilled water	carboxyl, hydroxy, aldehyde/ester groups responsible for reduction and stabilization	N/A	24 h/room temperature
	spherical/av.: 23.99 ± 8.43 nm	FTIR/SEM/XRD/EDX	antimicrobial/anti-biofilm agents	[280]

*Bacillus brevis*	not explicitly mentioned	amide (–CONH), hydroxy, and carbonyl corresponding to the protein (responsible for reduction)/C=C and –CH on protein responsible for stabilization	N/A	24 h/room temperature
	spherical/41–68 nm	AFM/SEM/TLC/FTIR	antimicrobial agents	[168]

*Enterobacter cloacae*	not explicitly mentioned	primary amide, secondary amide	N/A	72 h/room temperature
	spherical/12–30 nm	FTIR/SEM/XRD	antibacterial activity against MDR strains	[281]

EPS from *Leuconostoc lactis*	Milli-Q water	hydroxy groups	N/A	1 month/room temperature
	spherical/30–200 nm	SEM/TEM/XRD/UV–vis/AFM/TGA-DTA	textile industry: degradation of azo-dyes	[169]

*Pseudomonas stutzeri*	not explicitly mentioned	primary, secondary, and aliphatic amides	N/A	8 h/80 °C
	spherical/15–20 nm	TEM/FTIR	antibacterial activity against MDR strains	[170]

*Bacillus methylotrophicus*	not explicitly mentioned	extracellular enzymes and proteins	N/A	–/28 °C
	spherical/10–30 nm	FE-TEM/EDX/UV–vis	antibacterial activity	[282]

*Escherichia coli* (culture supernatant)	not explicitly mentioned	primary and secondary amines corresponding to the protein	N/A	24 h/room temperature
	spherical/40–90 nm (unoptimized condition) and 10–40 nm (optimized condition)	XRD/TEM	conductivity/ catalytic and antimicrobial activity	[283]

*Staphylococcus aureus*	Milli-Q water	extracellular enzymes	N/A	5 min/–
	varied morphology/160–180 nm	AFM/UV–vis	antimicrobial activity	[284]

cyanobacteria	Milli-Q water	–	N/A	60 min/30–60 °C
	spherical/60–80 nm	UV–vis/DLS/TEM	dye decolorization property	[285]

Yeast				

*Cryptococcus laurentii* (culture supernatant)	not explicitly mentioned	amide corresponding to peptides	N/A	48 h/28 ± 4 °C
	spherical/av.: 35 and 400 nm	UV–vis/TEM/XRD/FTIR	antifungal activity	[178]

*Saccharomyces cerevisiae*	not explicitly mentioned	cytoplasmic enzymes (oxidoreductase and oxidase) responsible for reduction/proteins responsible for stabilization	N/A	72 h/25 °C
	spherical/2–20 nm	TEM/DLS	cosmetics, foods, and consumer goods	[179]

Plant/fruit extracts				

green tea powder (*Camelia Sinensis*)	deionized water	tea polyphenols (responsible for stabilization)	N/A	15 min/60 °C
	spherical/34.68 ± 4.95 nm	UV–vis/XRD/FTIR/TGA/AFM/DLS/XPS	antibacterial activity	[286]

*Camelia Sinensis* extract	deionized water	not mentioned	N/A	30 days/room temperature
	one-dimensional (nanowires)/50 nm (diameter) and 1.3 µm (length)	FESEM/HRTEM/HAADF/UV–vis	antibacterial activity	[287]

blackberry fruit extract	deionized water	O–H and C=O groups	N/A	48 h/25 °C
	spherical/12–50 nm	TEM/FTIR/DLS/XRD/UV–vis	antioxidant activity	[288]

*Berberis Vulgaris* leaf extract	distilled water	not mentioned	N/A	1 h/room temperature
	spherical/30–70 nm	TEM/XRD/DLS/UV–vis	antibacterial activity	[187]

*Origanum vulgare* extract	deionized water	phytomolecules	N/A	2 h/85–90 °C
	spherical/2–25 nm	UV–vis/XRD/HRTEM/EDX/FTIR	antibacterial and antifungal activity	[186]

*Coffea Arabica* seed extract	deionized water	phenolic groups	N/A	2 h/room temperature
	spherical and ellipsoidal/20–30 nm	UV–vis/SEM/SEM-EDXA/FTIR/DLS	antibacterial activity	[289]

lignin	Milli-Q water	phenolic hydroxy groups	N/A	30 min/85 °C
	spherical/7.3 ± 2.2 nm and 14.3 ± 1.8 nm	UV–vis/TEM	not mentioned	[290]

tannin	not explicitly mentioned	hydroxy groups	N/A	2 h/50 °C
	one-dimensional (nanowires)/50 nm (diameter)	UV–vis/HRTEM/SEM/SAED/IR	detection of Pb(II) ions	[291]

clove oil	not explicitly mentioned	hydroxy groups from eugenol	N/A	not mentioned/room temperature
	one-dimensional (nanowires)/39 ± 0.01 nm (diameter) and 3 µm (length)	UV–vis/HRTEM/XRD/FTIR	conductive ink	[292]

apple extract (microwave-assisted)	ultrapure water	not mentioned	N/A	2.5 h for microwave-assisted reduction/room temperature and 96 h in thermally assisted one-pot reduction/100 °C
	spherical/28.24 ± 1.15 nm for thermally assisted and 22.05 ± 1.05 nm for microwave-assisted reaction	UV–vis/XRD/EDS	antibacterial activity	[293]

*Azadirachta indica*	not explicitly mentioned	C=O groups corresponding to alkyne groups, C–O and C–O–C bonds corresponding to flavonoids and terpenoids (responsible for stabilization)	N/A	15 min/room temperature
	spherical/34 nm	UV–vis/FTIR/TEM/DLS	antibacterial activity	[294]

*Elephantopus Scaber*	distilled water	O–H, C=O stretching of plant constituents and C=C stretching of aromatic rings	N/A	30 min/40 °C
	spherical/37.86 nm	UV–vis/TEM/XRD/FTIR	antimicrobial and anticancer activity	[255]

*Cinnamon zeylanicum*	dimethyl sulfoxide (DMSO)/ethanol/distilled water	aldehyde content (responsible for reduction)	N/A	2 h/60 °C
	spherical/2–10 nm (in ethanol), polygonal/5–25 nm (in distilled water), spherical/10–50 nm (in DMSO)	UV–vis/HRTEM	antibacterial activity	[295]

banana peel extract	distilled water	carboxyl, hydroxy and amide groups (responsible for reduction)	N/A	76 h/100 °C
	spherical/23.7 nm	UV–vis/EDX/XRD/SEM/TEM/FTIR	antibacterial activity	[296]

turmeric extract	Milli-Q water	hydroxy groups present in the curcumin powder and C–H bonds in turmeric powder responsible for the reduction	N/A	24 h/room temperature
	spherical/18 ± 0.5 nm	UV–vis/TEM/EDX/FTIR	antibacterial activity	[297]

marigold flower	ultra-purified water	N–H amid stretching, C–H stretching from vinyl disubstituted alkenes, and C–Cl stretching from alkyl halides (responsible for reduction)	N/A	24 h/room temperature
	varied (spherical, hexagonal, and irregular)/46.11 nm	UV–vis/XRD/FTIR/EDX/SAED/TEM	antibacterial activity	[298]

starch	Milli-Q water	O–H stretching (aliphatic hydroxy group) and C=O (responsible for reduction and stabilization)	N/A	48 h/25 °C
	spherical, polydispersed and amorphous/45.6 nm	UV–vis/TEM/SAED/XRD/FTIR/DLS	catalytic activity	[299]

ginger	deionized water	phenolic groups and flavonoids	N/A	2 h/room temperature
	spherical/10.10–18.33 nm	UV–vis/TEM	antioxidant and antimicrobial activity	[300]

Vitamins				

vitamin C (ascorbic acid)	high purity water	not mentioned	citrate	15 min/30 °C
	quasi-spherical/31 nm	UV–vis/TEM	not specifically mentioned	[301]

vitamin B2 (riiboflavin)	Milli-Q water	not mentioned	N/A	24 h/room temperature
	spherical/6.1 ± 0.1 nm, nanorods (10–20 nm diameter, 100–200 nm length)	UV–vis/TEM/SEM/EDX	catalytic polymerization of aniline and pyrrole	[204]

vitamin B12 (microwave-assisted)	Milli-Q water	not mentioned	N/A	3–6 min/100 °C
	irregular/70–600 nm	UV–vis/XRD/TEM/SEM	not specifically mentioned	[302]

Microalgae/algae				

*Chlorococcum humicola*	distilled sterile water	C–N bonds corresponding to aromatic and aliphatic amines (responsible for stabilization)	N/A	48 h/room temperature
	spherical/2–16 nm	SEM/TEM/FTIR/UV–vis/XRD	antibacterial activity	[278]

*Spirulina*	sterilized double-distilled water	O–H groups, C–O bonds corresponding to COOH group, and N–H bonds corresponding to primary and secondary amines	N/A	3,6,9 and 12 h/room temperature
	spherical/5–50 nm	UV–vis/FTIR/XRD/EDX/bio-TEM	antibacterial activity	[279]

*Chlorella Vulgaris*	not explicitly mentioned	C=O and –H (amide I and amide II) bonds corresponding to proteins (responsible for stabilization and reduction)	N/A	5 days/25 °C
	spherical/9.8 ± 5.7 nm	UV–vis/XRD/TEM/FTIR/EDX	antibacterial activity	[275]

*Padina pavonia*	not explicitly mentioned	N–H (amine), C–N (primary amines) groups and O–H groups (responsible for reduction)	N/A	3 h/room temperature
	spherical, triangular, rectangle, polyhedral and hexagonal/49.58–86.37 nm	UV–vis/TEM/DLS/FTIR	not specifically mentioned	[193]

*Chlamydomonas reinhardtii*	deionized water	amine and carbohydrate groups	N/A	192 h/room temperature
	spherical/5.6 ± 2.4 nm	UV–vis/XRD/TEM	not specifically mentioned	[192]

Viruses				

tobacco mosaic virus	not explicitly mentioned	hydroxy, carboxyl and thiol groups (responsible for reduction)	N/A	1 h/50 °C
	spherical/2 nm	UV–vis/TEM	catalytic and antibacterial activities	[176]

Fungus				

*Rhizopus stolonifer*	deionized water	carbonyl group of amino-acid residue and peptide-protein	N/A	2 days/40 °C
	spherical/2.86 ± 0.3 nm	UV–vis/XRD/HR-TEM/FTIR	not specifically mentioned	[303]

*Penicillium aculeatum*	Milli-Q deionized water	N–H and C=O from the amide group of proteins, –C–N belonging to aromatic and aliphatic amines	N/A	4 weeks/room temperature
	spherical/4–55 nm	HRTEM/XRD/FTIR/UV–vis	antibacterial activity	[174]

*Penicillium chrysogenum*	deionized water	–O–H, C–, C–O–, COO–, and –N–H stretching associated with proteins	N/A	–/28 °C
	spherical/9–17.5 nm	FTIR/TEM/DLS	antifungal activity and ability to prevent mycotoxin production	[172]

*Fusarium oxysporum*	deionized water	not mentioned	N/A	–/37 °C
	spherical/1–50 nm	SEM/TEM	antibacterial activity	[171]

Sugars				

brown sugar	distilled water	C–C and C–O corresponding to sucrose, glucose and fructose	N/A	2 h/50 °C
	varied (spheres, cubes, and bars)/–	UV–vis/TEM/HRTEM/EDX/FTIR/MS	biomedical and pharmaceutical applications	[199]

white sugar	double-distilled water	–OH groups corresponding to gluconic acid	N/A	10 min under sunlight, 2–3 h without sunlight/room temperature
	spherical/10–25 nm	UV–vis/FTIR/NTA/TEM	biomedical and pharmaceutical applications	[200]

#### 3.1 Bacteriogenic synthesis

Bacteriogenic synthesis is a fairly advantageous process towards facile synthesis of AgNPs due to the reactive response of bacteria to silver. The Ag-resistant bacterial strains are utilized for the synthesis of AgNPs, as they can accumulate Ag atoms on the cell walls [104]. The bacteriogenic synthesis process can be either extracellular or intracellular. AgNPs are synthesized either by the biomass [267] or the cell culture supernatant [259]. AgNPs synthesized using bacteriogenic pathogens are commonly spherical in morphology and range from 5–200 nm in size. The mechanism through which AgNPs are synthesized is still not well understood; however, Fourier-transform infrared (FTIR) spectroscopy results from previous studies suggest that carboxylic and hydroxylic groups, in addition to primary and secondary amides corresponding to cellular proteins and enzymes, are responsible for the synthesis and stabilization of AgNPs (Table 2). These components are present in both the biomass and the cell culture supernatant. It was previously demonstrated that the synthesis rate can be faster for some bacterial strains including *Escherichia coli* (*E. coli*) and *K. Pneumonia* when the cell culture supernatant is used [268]. However, the main downside of bacteriogenic synthesis is the slow synthesis rate and large size distribution compared to other green methods [104,259]. The applications of bacteria-synthesized AgNPs can be seen in the development of antimicrobial agents, biosensors, optics, solar energy, and drug delivery [267–269].

#### 3.2 Fungi-mediated synthesis

Fungal species have demonstrated significant potential for the synthesis of AgNPs. Their high binding and bioaccumulation capacity, intracellular uptake, and ease of handling provide them with additional advantages compared to bacteria [260]. Previous studies have shown that fungi-synthesis processes are followed by an enzymatic process, affecting the formation of stable AgNPs in the range of 5–15 nm [270]. However, this range can vary with respect to reaction conditions. FTIR results show that similar to bacteria, carbonyl, amide, and hydroxy groups corresponding to the cellular protein are responsible for the synthesis and stabilization of AgNPs [178–179]. The less non-pathogenic behavior of fungi and their faster synthesis rate suggest their use over bacteria. Fungi-synthesized AgNPs have proved to have noticeable anti-bacterial activity. Naqvi et al. showed that synthesized AgNPs using *A. flavus* fungi increased the biocidal effectiveness against drug-resistant bacteria significantly [271]. Fungi-assisted synthesis has proved to be a promising approach towards the production of AgNPs; however, the pathogenic behavior of the fungi and long synthesis periods compared to other green methods makes it inferior among green synthesis processes.

#### 3.3 Plant virus template-mediated synthesis

Plant-based viruses as biotemplates have been rarely used for the synthesis of silver nanostructures compared to other methods. To the best of our knowledge, there have been only a few studies [176,261–263] that have used plant viruses to synthesize silver nanostructures. In particular, *tobacco mosaic virus* (TMV) is used as the most common template to produce rod-shaped Ag nanostructures. However, TMV is not the only template that can be used for the synthesis of Ag nanostructures. Among others, *Brome Mosaic Virus* (BMV), *Cowpea Chlorotic Mottle Virus* (CCMV), *Cowpea Mosaic Virus* (CPMV), *Hibiscus Chlorotic Ringspot Virus* (HCRSV), *Red Clover Necrotic Mosaic Virus* (RCNMV), and *Turnip Yellow Mosaic Virus* (TYMV) may also be used for the synthesis of Ag nanostructures [272]. The AgNPs can be synthesized either inside the viral template, within the interface, or on the outer surface [272]. In one of the early works, Dujardin et al. [261] synthesized AgNPs in cylindrical matrices using TMV as the biotemplate. In this work, AgNPs were 2–4 nm in size and it was shown that AgNPs, unlike platinum and gold nanoparticles, were coated on the inner surface of the TMV channel. The synthesis process is known to be mediated by the amino acid functional groups [263]. Although viral templates have not been investigated as much as other approaches, viral template-mediated Ag nanostructures have been demonstrating promising potential in targeted imaging and therapeutic delivery systems [272]. In addition, they can be used to synthesize 1D Ag nanostructures [262–263]. This characteristic is obtained due to the rod-shape morphology of some plant viruses such as TMV. The synthesis of AgNPs using 1D templates can facilitate their application as bio-semiconductors [263]. One of the main advantages of viral templates is the simple synthesis of small AgNPs [261–262]. However, their major drawback is the lack of strong metal-binding sites along the biotemplate surface [262]. In addition to that, the preparation of the viral templates is time-consuming and multiple coating cycles may be required to yield a uniform coating of metal nanostructures on their surface. We have recently reviewed engineered TMV and its virus‐like‐particles (VLPs) for synthesis of biotemplated nanomaterials. We also discussed the recent advances on novel *barely stripe mosaic virus* (BSMV) and its VLP as a novel template for metal nanoparticle synthesis [273–274].

#### 3.4 Algae-mediated synthesis

The synthesis of AgNPs using marine-based microorganisms has emerged as one of the novel and promising routes due to their non-toxicity and eco-friendly nature [189,275]. Cyanobacteria, brown algae, and green algae are the most common types of algae used for the synthesis of AgNPs [189]. For most applications, the single-cell type of the organism known as microalgae is used to synthesize AgNPs [191]. This enables formation of homogeneous microalgal suspensions which can be used directly for the synthesis process. In the synthesis process, the microalgal biomass in the aqueous phase, cell-free aqueous extract, aqueous supernatant of dried algae, or aqueous filtrate of the broth are mixed with the silver solution (mostly silver nitrate) to synthesize AgNPs [189]. The synthesis process is intracellular when the reaction takes place within the cells, and extracellular when biosynthesis occurs outside of cells due to the presence of biomolecules [191], which depends on the type of cell culture used. For instance, cell-wall deficient cells are typically more inclined towards intracellular biosynthesis as the cell-wall is known to act as a barrier for the diffusion of metal cations into the cytoplasm [276]. When synthesized, the AgNPs are capped by a matrix of polysaccharides in and out of the cells [191], and the size of synthesized AgNPs varies depending on the cell type; however, AgNPs with average sizes as low as 4.3 nm [192], and as high as 35 nm [277] were reported. The synthesized AgNPs are typically spherical in shape [192,278–279]; however, varied morphologies were also reported [193]. Among the applications, algae-mediated AgNPs have shown effective antioxidant and antibacterial activity [264,275]. The advantages of the algae-mediated synthesis process are low reaction temperatures, use of non-hazardous reagents, and synthesis of relatively small particles with uniform morphology. However, the main disadvantage of this type of biosynthesis is the significantly low production rate [264–265].

#### 3.5 Plant/plant-extract-mediated synthesis

The synthesis of Ag nanostructures using plants and plant extracts has recently gained more attention. These methods can act as appropriate alternatives for other methods as a result of their simplicity, low-cost, non-toxicity, and simple scale-up capability [186]. In addition, thanks to their non-pathogenic and biocompatibility characteristics, AgNPs synthesized by plants and plant extracts are ideal for biomedical applications [304]. Plant extracts contain phenolic compounds such as flavonoids and alkaloids which are soluble in water [305]. These compounds provide the reagent with unique reducing and capping characteristics [306]. This can be proved by FTIR observations, where polyphenols are a major common functional group responsible for reduction of Ag ions and stabilization of AgNPs (Table 2). The AgNPs can be functionalized with respect to the type of plant or plant extract reagent and reaction conditions [307]. Plants are a natural source for the removal of heavy metals from soils and underground water [308]. The removal of heavy metals takes place by phytoremediation strategies such as phytoextraction, phytofiltration, phytostabilization, phytovolatilization, phytodegradation, rhizodegradation, and phytodesalination [309]. A variety of plants such as *Noccaea caerulescens*, *Pteris vittata*, and *Sedum plumbizincicola* were previously demonstrated to have substantial heavy-metal detoxification capability [310–312]. One of the most significant factors in the detoxification process is the redox potential [313], which is seen as an opportunity to utilize plants and their components for the reduction of metal cations and synthesis of metal nanoparticles such as AgNPs. The synthesis of AgNPs by plants and their components may be categorized into in vivo and in vitro synthesis processes [140]. The in vivo synthesis refers to synthesis of AgNPs inside the plants and in vitro synthesis refers to synthesis by components extracted from the plants. In the first in vivo synthesis study, Torresdey et al. [314] synthesized AgNPs (spherical, 2–20 nm in diameter) using *Alfalfa Sprouts*, where it was reported that silver (Ag^0^) was absorbed from the agar medium through the roots and transferred into plant shoots. It was also reported in this work that the nucleation and formation of AgNPs occur within the plant tissue. However, it is debated by other studies whether the Ag^+^ ions are reduced outside or inside of the plant [140,315]. Later, several studies reported the synthesis of AgNPs using *Brassica Juncea* [316–319], in which they reported the presence of AgNPs in plant biomass. It is shown that the major compounds for the synthesis of AgNPs are phytochemicals that naturally exist in plants including flavones, terpenoids, catechins, and polyphenols [183,315,320], which may also include carboxylic acids, ketones, and aldehydes functional groups. Many studies have reported synthesis of AgNPs by in-plant phytochemicals in the water-soluble form [321]. This is advantageous because the water-solubility of the phytochemical compounds simplifies the process. Various parts of plants such as roots, fruits, seeds, needles, and aerial parts may be used for extraction of phytochemicals [321]. These extracts contain a substantial amount of polyphenols which are strong antioxidants [140,186] with significant redox potential. A comprehensive mechanism for the synthesis of AgNPs by plant extracts has not yet been proposed; however, the reduction mechanism may be explained by identifying the responsible functional groups. Makarov et al. [322] hypothesized that when flavonoids, a large family of polyphenols, are used as reducing agents, reactive hydrogens are released via a tautomerization process, in which flavonoids are transformed to the keto-form, which leads to reduction of Ag^+^ to Ag^0^. In addition, hydroxy (–OH) groups are known to be a prominent functional group for reduction of Ag^+^ [321]. The concentration of the polyphenols varies depending on the plant from which they were extracted [321]. Therefore, the properties of the synthesized AgNPs, such as size and morphology, can be tuned by selecting the plant source, and adjustment of extract composition [180,323].

The plant-mediated synthesis of AgNPs is fairly simple. The reducing agents used for the synthesis of AgNPs are used in the form of soluble powder which was previously extracted, or obtained through a common extraction procedure [183]. For synthesis, the extract solution is simply mixed in an aqueous environment with the silver precursor such as AgNO_3_ and maintained at a desired temperature. A prominent advantage is that these reactions can be performed at room temperature, and successfully result in formation of AgNPs, which is reported by many studies [187,287,289,292–294,297–298,300]. However, the characteristics of the AgNPs, such as size and morphology, are highly sensitive to the reaction conditions. Parameters that dictate such characteristics are extract composition and concentration, temperature, pH, Ag^+^ concentration, reaction duration, and stirring rate [183].

The number of studies using plant extracts as reducing agents has been recently growing and various types of plant extracts have been used as reducing, capping, and stabilizing agents for the synthesis of AgNPs. Johnson et al. [324] synthesized AgNPs using *Odontosoria Chinensis* extract and reported the formation of spherical AgNPs with diameters ranging from 22.3 to 48.2 nm. The reaction occurred at 40 °C, and a complete reduction of Ag^+^ after 10 min was reported. Agglomeration was observed in the case of temperatures higher than 40 °C. Also, the most optimal concentration ratio was reported to be at 1:9 (extract/AgNO_3_). They reported that carboxylic acids and hydroxy functional groups were mainly responsible for the synthesis of AgNPs, and the presence of terpenoids, tannins, polyphenols, and steroids in *Odontosoria Chinensis* extract was confirmed before the synthesis experiments. The synthesized AgNPs demonstrated successful anti-inflammatory and antidiabetic activities, which was due to polyphenols, terpenoids, and tannins in the extract in addition to the AgNPs themselves. Sivakumar et al. [325] synthesized spherical AgNPs with an average size of 10.3 ± 1.7 nm using *Parthenium hysterophorus* aqueous extract. The aqueous extract was mixed with a 10 mM AgNO_3_ solution with a volumetric ratio of 3:100. The mixture was incubated in the dark for 1 h. FTIR characterization results showed the presence of polypeptides, amine, germinal methyl, and hydroxy groups as functional groups responsible for reducing Ag^+^ ions and formation of AgNPs. The synthesized AgNPs then demonstrated significant antibacterial activity against *E. coli*, *P. auriginosa*, *B. subtillis*, *S. aureus*, *E. feacalis*, and *K. pneumonia*, and anticancer activity against HepG2 cell lines. It was later demonstrated by Nouri et al. [326] that synthesis using *Mentha Aquatic* can result in the formation of small AgNPs with relatively narrow size distribution. In their study, *Mentha Aquatica* leaf extract was mixed with AgNO_3_ solution at different reaction conditions. The most optimal reaction condition was reported to be at a pH of 9.5, temperature of 90 °C, concentration ratio of 1:1, and 60 min reaction time. However, they showed that ultrasonication can reduce both the reaction time and particle size. Without ultrasonication, the average size was observed to be 14 nm, while with ultrasonication, this size was reduced to 8 nm. In addition, the increase in ultrasonication power was proportional to the decrease in reaction time. The reaction time decreased from 60 min to 10 min by increasing the ultrasonication power from 0 to 200 W. This was attributed to the enhanced diffusion of compounds due to sonic waves. In another study, Tanase et al. [327] synthesized spherical AgNPs using aqueous bark *Picea abies L*. extract. The reaction was performed at 70 °C and pH 9 with different AgNO_3_ and extract concentrations. Although colloidal AgNPs were synthesized, the size and size distribution were significantly high (100–500 nm). The most optimal scenario was attributed to 1 mM of AgNO_3_ with a volumetric ratio of 1:10 (extract/AgNO_3_) and a reaction time of 3 h. The results demonstrated that higher AgNO_3_ concentration results in inhibition of the synthesis process. The responsible functional groups were confirmed by FTIR to be aldehydic, carbonyl, and hydroxy groups of phenols and carboxylic acids. The AgNPs showed strong antibacterial activity against *E. coli* and Klebsiella pneumonia, comparable to the antibacterial activity of gentamicin.

Most plant extract synthesis studies use extracts as both reducing and capping agents at the same time. However, this might not be advantageous in some cases. Ranoszek-Soliwoda et al. [188] synthesized spherical AgNPs using cacao beans and grape seed extract and investigated the effect of sodium citrate with various molar ratios with respect to AgNO_3_ solution (1.5–6 for sodium citrate, and 1.5–9 for the extract). Acetone and ethanol were used as a solvent for cacao beans and grape seed extract, respectively. The initial concentration of both reagents was set to 2 wt %, and the concentration of AgNO_3_ was kept constant at 1 wt %. The mixtures were heated for 15 min and then cooled down to room temperature. Using various molar ratios, they reported that cacao bean extract resulted in aggregation of AgNPs followed by sedimentation, and therefore, it cannot be used as reducing and stabilizing agents at the same time. However, monodisperse AgNPs with an average size of approximately 15 nm were obtained using sodium citrate as the capping agent. Under these conditions, the molar ratio did not have a significant effect on the size of AgNPs, and their size was maintained between 11–15 nm at different molar ratios of cacao bean extract and sodium citrate. The reason was attributed to the fact that sodium citrate is able to form stable complexes with the polyphenols present in the extract, which then acts as reducing and capping agent. This was nevertheless different for grape seed extract. Without the presence of sodium citrate, AgNPs agglomerated and had a large size distribution, but in the presence of sodium citrate, the size was maintained at 10–18 nm for different sodium citrate molar ratios, and the size distribution was smaller. Furthermore, it was reported by Mohaghegh et al. [328] that sodium citrate can form complexes with Ag^+^ at ambient temperature, and act as reducing and capping agent at the elevated temperature. In another study, Ranoszek-Soliwoda et al. [329] reported the formation of citrate–tannic acid complexes which were responsible for reduction and stabilization of tannic-acid-mediated AgNPs. They also stated that the mere use of sodium citrate could result in inhomogeneous sizes and a broad size distribution. To the best of our knowledge, it has not yet been well determined what type of plant extracts can be used as reducing and capping agents at the same time. The formation of silver nanoparticles by plant extracts depends highly on the plant extract composition along with various process parameters such as pH, temperature, and concentration ratio.

**3.5.1 Effect of pH.** The pH is known to be a significant parameter in controlling the size, morphology, and stability of AgNPs [330]. Singh et al. [331] synthesized spherical AgNPs using *Hibiscus* leaf extract ranging 12–17 nm in diameter. Temperature and pH were reported to be the most significant factors in controlling the synthesis process. The effect of pH was mainly investigated in this work and it was observed that at pH 3 the AgNP formation process stops as there was not any further color change, and at pH 10 agglomeration occurs upon the addition of the AgNO_3_ solution. At pH 6 however, the reduction process started upon the addition of AgNO_3_ and was observed for 30 min. The most optimal condition to minimize the size of AgNPs was reported to be at a pH of 6, temperature of 70 °C, 30 min of reaction time, and 5 mM AgNO_3_ solution (1:1.5 volumetric ratio with respect to the extract). Their FTIR studies demonstrated that the hydroxy functional groups and polysaccharides were responsible for reduction and stabilization of AgNPs. In another work by Handayani et al. [332], it was demonstrated that alkaline conditions are more favorable for the synthesis of smaller nanoparticles. They synthesized AgNPs using *Pometia pinnata* (*Matoa*) leaf extract for 24 h with 1:2 volumetric ratio (extract/AgNO_3_). They demonstrated that at pH 11, the AgNPs were 10–50 nm in size and of spherical and hexagonal morphologies, while at pH 4 the AgNPs were observed to be 50–80 nm with spherical and triangular morphologies. It was also reported that a change in pH affects the reducing capability of the extract, which is also confirmed by other studies [333–334]. The pH is also reported to control the zeta potential [330,332] and results in particle stability adjustments [330]. The higher the value of the zeta potential, the stronger the long-term stability of the AgNPs, thereby preventing their agglomeration [330,335]. Alkaline pH also has the potential of increasing the reaction rate due to the enhanced deprotonation of phenolic compounds in basic condition [336]. In addition, alkaline pH enables more –OH to take part in the reduction reaction, thus increasing the reduction strength [337].

**3.5.2 Effect of temperature.** Temperature is another crucial factor in controlling the size and morphology of AgNPs. Increasing the temperature equals increasing the reaction rate which is favorable for rapid synthesis. However, high temperatures (more than 60 °C) may result in denaturation of the extract compound, and thus alter their reduction potential and result in a significant increase in particle size and even agglomeration [140]. The extract compound should therefore be carefully selected and tested in order to avoid denaturation. Madivoli et al. [338] synthesized AgNPs using *Lantana trifolia* extract by changing the temperature from 20 to 35 °C. It was shown that a decrease in temperature resulted in a broader surface plasmon peak, attributing to a larger size distribution contrary to higher temperatures. However, increasing the temperature resulted in the formation of larger particles, 60 nm in the case of 35 °C and 48 nm in the case of 20 °C. Nevertheless, AgNPs with a size of 37 nm were obtained at 30 °C. Therefore, there was a sharp increase in particle size by changing the temperature from 30 to 35 °C. In addition, in a study performed by Anbu et al. [339], AgNPs were synthesized at 50 °C and were slightly larger compared to those synthesized at 37 °C. The increase in particle size due to an increase in temperature was also confirmed by previous studies [307,340–342]. Furthermore, Liu et al. [343] used *Cinnamomum Camphor* leaf extract to synthesize AgNPs. They demonstrated that an increase in temperature will result in an increase in particle size under sufficient Ag^+^ ions (Ag^+^ concentration equivalent or excess to the extract concentration), and results in a decrease in particle size under insufficient Ag^+^ ions (extract concentration excess to Ag^+^ concentration). They concluded that under sufficient Ag^+^ ions, particle size is not affected by nucleation, but rather by growth. They proposed on the contrary, that under insufficient Ag^+^ concentration, all the Ag^+^ ions are consumed rapidly through a burst nucleation process, thus not allowing further growth. It can therefore be concluded that temperature and concentration are both significant factors and have an interactive effect on controlling the morphology and size of AgNPs.

**3.5.3 Effect of concentration/concentration ratio.** The concentration of plant extract and silver precursor, as well as their ratio, play a major role in controlling the size and morphology of AgNPs. Hasnain et al. [344] synthesized spherical AgNPs using purple heart plant leaf extract and investigated the effect of various parameters using the design of experiments (DoE) approach. Their results indicated that an increase in AgNO_3_ concentration (from 0.01 to 0.1 M) at constant purple heart plant extract volume (1.4 mL) and relatively lower temperatures (60 °C), resulted in a decrease in particle size (from ≈120 to 100 nm). They also observed an initial decrease (from ≈310 to 150 nm) followed by a sharp increase in particle size (from ≈150 to 335 nm) at higher temperatures (80 °C). In addition, an increase in AgNO_3_ concentration at constant temperature (80 °C), and relative to various volumes of purple heart plant extract, resulted in an initial decrease (from ≈310 to 150 nm) followed by an increase in particle size (from ≈150 to >350 nm). However, changing the purple heart plant extract volume did not have a significant effect on the particle size. Therefore, the AgNO_3_ concentration had a much greater effect on determining particle size. Also changing the temperature from 60 to 80 °C resulted in an approximately linear and continuous increase in particle size (from ≈100 to 330 nm), meaning that temperature was a stronger parameter compared to plant extract concentration. Furthermore, Htwe et al. [345] synthesized AgNPs with quasi-spherical morphology using *Imperata* cylindrical plant extract as the reducing agent and ascorbic acid as the capping agent. They showed that increasing the AgNO_3_ concentration (from 0.5 to 0.9 mM), at constant extract and ascorbic acid concentration, resulted in increasing the particle size (from 32.7 to 39.9 nm). Their ultraviolet–visible (UV–vis) spectroscopy results also showed that at 0.5 mM AgNO_3_, the absorption peak was narrower compared to 0.7 and 0.9 mM AgNO_3_, indicating lower size distribution. Khan et al. [346] utilized *Piper Betle* leaf aqueous extract to synthesize AgNPs. They investigated the effect of concentration on the size and stability of AgNPs by testing various AgNO_3_ concentrations (1, 2, 3, and 4 mM) and extract concentrations (1:2, 1:4, and 1:8 dilution ratio with respect to the crude extract). They reported the most optimal conditions to be at 2 mM of AgNO_3_ and 1:4 dilution ratio of the extract. Their results indicated that increasing AgNO_3_ concentration leads to shifting the UV–vis peak to the higher wavelengths indicated formation of larger nanoparticles. Aggregation was also observed for higher AgNO_3_ concentrations. They also reported that higher concentrations of the extract could result in instability and aggregation of AgNPs, which is also confirmed by a previous study [347].

### 4 One-dimensional silver nanostructures and their green synthesis

One-dimensional (1D) silver nanostructures are characterized as a collection of silver atoms in a one-dimensional pattern. They range from tens to a few hundreds of nanometers in diameter, and their length is of the order of tens of micrometers. 1D silver nanostructures could either be silver nanowires (AgNWs) or silver nanorods (AgNRs). These two are distinguished by the aspect ratio, which is the ratio of the length to the diameter. Typically, 1D silver nanostructures with an aspect ratio of 10 or higher are considered as AgNWs while those with aspect ratios lower than 10 are considered as AgNRs [90]. In general, 1D metallic nanostructures have unique plasmonic properties as they can uniquely interact with light because of their plasmonic characteristics [348–349]. AgNWs, in particular, possess great surface plasmon resonance (SPR) characteristics, high conductivity, and remarkable flexibility in addition to having a simple fabrication process [350]. The synthesis of AgNWs has been under progressive investigation during the last decade due to their broad range of applications including nanoelectronics.

AgNWs have been widely applied in electronics. In particular, there have been promising developments in transparent conductive electrode (TCE) manufacturing for next generation touchscreens by applying AgNWs [351–354]. To name a few, C3Nano^®^, Cambrios^®^, and 3M^®^ are among the companies that have been developing AgNW-based inks for TCEs used in the flexible touchscreens manufacturing. Currently, indium tin oxide (ITO) is still the dominant material used in TCEs due to its high transmittance and low sheet resistance [67,355]. Its high conductivity and transmittance level made ITO a fairly appropriate candidate for TCEs; however, weak flexibility, limited supply, costly coating process, highly expensive large-scale manufacturing, and the cost of the material itself have limited a potentially broader application in optoelectronics [67,90]. As a result, ITO-embedded TCEs are gradually giving their position to AgNW-embedded TCEs. One of the most important aspects of the synthesis of AgNWs is to precisely control their size and morphology to achieve a uniform high aspect ratio that strongly affects their optical and transmittance properties [356–358]. Achieving a uniform high aspect ratio depends on the synthesis process and the reaction conditions [357]. As a result, selection of the reducing and stabilizing agents, and identifying the most important factors that control the synthesis process are essential in determining the length and diameter of the synthesized AgNWs.

There are generally two methods for the synthesis of AgNWs, which are hard template and soft template methods. In hard template methods, AgNWs are produced using cylindrical nanoporous structures such as carbon nanotubes (CNTs) [359]. On the other hand, in soft template methods, capping agents such as polyvinylpyrrolidone (PVP) or cetyltrimethylammonium (CTAB) are used to direct the growth of initially formed silver seeds to AgNWs [349]. Hard template methods have several disadvantages compared to soft template ones. The yield of AgNWs is significantly low and the templates are very hard to remove [90]. On the other hand, the template removal issue is nonexistent in soft template methods and soft templates can be used in the solution phase. Block copolymers [360] and polyvinyl alcohol (PVA) [361] are among the other soft templates used for the synthesis of AgNWs. Currently, the most common method for the synthesis of AgNWs is through the polyol process, in which ethylene glycol is usually used as the solvent and reducing agent, PVP as the capping agent, and in the presence of a small amount of salt mediator. This method is being used to synthesize AgNWs in large commercial scales and has proved to be simple and able to provide relatively proper control over morphology and aspect ratio [362]. The synthesis of AgNWs by the polyol approach was first introduced in 2002 by Xia’s group [363]. In early studies, the synthesis was performed using Pt seeds to promote nucleation and growth of AgNWs. In order to make the process more economical and simpler, while maintaining a high yield and aspect ratio, further modifications were later applied in the synthesis process, such as the gradual addition of AgNO_3_ solution using syringe pumps [364], using salt mediators such as Fe [365] and Cu [366] metal salts, and increasing preheating time [357]. The combination of exceptional size and morphology-dependent chemical, optical, and physical characteristics of 1D Ag nanostructures with their potential antibacterial activity, is evidence of a critical need to develop synthesis processes appropriate for their industrial-scale applications. These synthesis processes must meet some criteria including being scalable, low cost, and low toxicity of the materials involved in the synthesis process. These criteria are in excellent agreement with the goals in the development of green and sustainable synthesis of metal nanostructures. Polyol synthesis has a fairly high energy consumption due to high temperatures (>120 °C). In addition, in a more recent polyol processes, a 1 h preheating time for ethylene glycol was required, which adds to the experiment duration at relatively high temperatures. While progress with improving the polyol process is ongoing, some studies started to use green reagents for the synthesis of AgNWs. We have recently reviewed the polyol silver nanowire synthesis and its outlook for a green process [367]. In 2009, Tian et al. synthesized single-crystalline AgNWs – 25 nm in diameter and 20 µm in length – using tannin acting as both reducing and capping agent [368]. Tannin is a natural polyphenolic compound found in various plant extracts, such as oak barks, galls, and teas. They can act as strong reducing agents due to the oxidization of –OH functional groups in their molecular structure [369]. The AgNWs were synthesized at room temperature, and it was found that an increase in the tannin concentration resulted in AgNWs with a lower aspect ratio. They reported that the slow reduction rate could favor the formation of twinned silver seeds with decahedron morphology. On the contrary, in a more recent work, Dong et al. reported that at low temperatures, tannin molecules cannot selectively adsorb on the {100} facets of AgNWs due to the lack of driving energy [291]. In this work, AgNWs were synthesized at 50 °C for 2 h with the same AgNO_3_ and tannin concentrations used in Tian et al.’s study. The yield of AgNWs in this work was reported to be much higher (as high as 90%) compared to Tian et al.’s study (5–10%). The AgNWs were reported to be 60 nm thick in diameter and several tens of micrometers long. Although low temperatures were considered to be unfavorable for the growth of AgNWs, higher temperatures (higher than 50 °C) were not favorable either, which is due to the fact that tannin molecules do not have adequate time to adsorb on the specific facets, thus resulting in formation of nanoparticles. They also reported that decreasing the pH from the basic to acidic region results in increasing the yield of AgNWs, with pH 5 being the optimal value. In another study, Lin et al. synthesized AgNWs using *Cassia fistula* as both reducing and capping agent [370]. The reaction was performed at room temperature for 48 h, and the synthesized AgNWs were reported to be 50–60 nm in diameter and tens of micrometers in length. They reported that the Ostwald ripening process was indispensable to the accumulation of smaller nanoparticles into AgNRs and ultimately AgNWs. They also reported that increasing the temperature would result in the formation of mere nanoparticles due to the alteration of interaction between the biomolecules and certain facets. Jeevika et al. used clove oil, as both reducing and capping agent to synthesize AgNWs with a diameter of 39 nm and length of 3 µm [292]. Clove oil is rich in eugenol, which is a phenolic compound. The reaction was carried out at room temperature and –OH functional groups from eugenol were found to be responsible for the synthesis of AgNWs. Eugenol molecules were also responsible for stabilizing AgNWs, as they can bind to the initially formed Ag seeds’ facets and direct the growth. Soleimani et al. synthesized AgNRs with sharp and blunt ends using *F. oxysporum* as a reducing agent and starch as a capping agent [371]. In their work, the reaction was carried out at 30 °C for 3 days, with the pH adjusted at 3. For AgNRs with sharp ends, the average diameter and length were 181 nm and 4.3 µm, and for AgNRs with blunt ends, the average diameter and length were 204 nm and 3.9 µm, respectively. They reported that the initially formed Ag nucleus was single-crystal, and starch was able to cover the {100} facets of the NRs. It is also noteworthy to mention that other morphologies were obtained by changing the concentration of AgNO_3_, temperature, pH, and reaction time. In another study, Flores-González et al. used *Camellia Sinensis* extract in presence of a small concentration of PVP to synthesize AgNWs with dimensions of 50 nm in diameter and 1.3 µm in length [287]. *Camellia Sinensis* is a strong phenolic compound with significant antioxidant potential. The reaction was carried out at room temperature for 30 days under dark conditions. They reported that PVP was able to selectively adsorb to {100} and {111} facets, thus directing the growth of AgNWs. They also explained that the Ostwald ripening process resulted in attraction of the previously formed AgNPs, therefore leading to NW growth. However, it is important to note that at the beginning of the reaction, PVP was added to the reaction in the form of powder, and the temperature of the solution was kept at 45 °C for complete dissolution of the PVP. Nadagouda et al. used vitamin B2 to synthesize AgNWs and AgNRs at room temperature for 24 h [204]. In their study, AgNWs with diameters ranging from 10 to 20 nm and lengths of several hundreds of micrometers were obtained when isopropanol was used as the solvent, and AgNRs structures were obtained with a thickness of 100 to 200 nm and lengths of tens of micrometers when acetone and acetonitrile were used as the solvent. Hemmati et al. obtained AgNRs using brown sugar as both reducing and capping agent [199]. The AgNRs were around 50 nm thick in diameter and a few micrometers long. The reaction was performed at 50 °C for 2 h.

The main advantage of the green synthesis of 1D Ag nanostructures is the fact that the selected green reagent can act as both capping and reducing agent at the same time, therefore triggering and continuing the reduction process while controlling growth. As a result, providing an additional capping agent such as PVP may not be required, which could reduce the overall material cost, since green reducing agents such as plant extracts are not expensive. Another important advantage is the possibility of performing the synthesis experiments at significantly lower temperatures compared to the polyol process, where temperatures of 120–200 °C are required. Some reactions can even be carried out at room temperature. This is quite beneficial in reducing energy consumption and risk, thus making the large-scale synthesis processes more cost-effective and safer. Another advantage is the fact that synthesis experiments may be performed without using exotic seeds and metal salts. Green reagents such as tannin and clove oil have shown that the synthesis of AgNWs with high yield and aspect ratio is possible. As a result, if the responsible factors are identified properly, high aspect ratio and yield can be achieved, comparable to those achieved through the polyol process.

### 5 Effect of reaction parameters on the green synthesis of 1D Ag nanostructures

The effect of reaction parameters such as pH, temperature, reducing agent concentration, and silver precursor concentration is crucial for the formation of 1D silver nanostructures such as AgNRs and AgNWs. To facilitate the growth of 1D nanostructures, the reaction conditions should be favorable to initiate an anisotropic growth of the seeds in a kinetically controlled synthesis process by adjusting and controlling the generation and deposition rate of silver atoms [372]. When the number of atoms for heterogeneous nucleation is lower than the growth sites available on a seed surface, the seed is expected to undergo an anisotropic growth [373]. There are different ways to make this process possible. For instance, applying a relatively low temperature (i.e. room temperature) can result in the suppression of adatomic diffusion, and consequently results in an anisotropic growth process [373]. However, this also depends on the reducing capability of the reagent at room temperature as well as its concentration, reaction pH, and metal precursor concentration. For instance, tannic acid, which is a hydrolysable form of tannin, has been used for the synthesis of both AgNPs and AgNWs [368,374–375]. Tannic acid has the potential to be hydrolyzed into glucose and gallic acid in weak basic/acidic conditions at room temperature [376–377]. Gallic acid can serve as a reducing agent while glucose serves as a stabilizing agent [378]. This promising dual property of tannic acid makes it usable as both reducing and capping agent. The reducing capability of gallic acid increases by increasing the pH [378]. In the case of AgNP synthesis at room temperature and basic pH using tannic acid, gallic acid plays its role as a strong reducing agent while glucose acts as a strong stabilizing agent [379–380]. However, in the case of AgNW or AgNR synthesis at room temperature using tannic acid, the acidic pH is considered to be more favorable due to the slower reduction rate provided by gallic acid [375]. Three studies agree on the fact that acidic pH is more favorable for the growth of 1D nanostructures in the presence of tannic acid [291,368,375]. However, higher temperatures may not be favorable for anisotropic growth due to highly rapid nucleation. Two studies have considered the room temperature conditions to be more favorable for the growth of AgNWs in acidic pH and the presence of tannic acid [368,375]. However, only one study by Dong et al. reported slightly higher temperatures (50 °C) to be more favorable for the formation of AgNWs [291]. Although they mentioned that this is due to difference in facet absorption energy at different temperatures, a clear and deep explanation remains disputed as to why this difference occurs. The study on the effect of pH and temperature on the yield of 1D silver nanostructures using other green reagents remains limited.

The concentrations of the reducing agent and silver precursor are other important factors in controlling the yield of 1D Ag nanostructures. Yi et al. investigated the effect of tannic acid concentration on the yield of 1D nanostructures by changing its concentration from 5 to 1 mM while keeping the AgNO_3_ concentration at 3 mM [375]. They demonstrated that the yield of 1D nanostructures such as nanorods increased as the tannic acid concentration decreased to 1 mM. This is also in accordance with the study by Tian et al. where they used a 1 mM tannic acid concentration [368]. This was attributed to the sufficiently slow reduction process for the formation of single crystal nanowires. However, the mere effect of AgNO_3_ concentration on the yield of 1D silver nanostructures was not investigated.

Considering the knowledge gap in the green synthesis of 1D Ag nanostructures, it is essential to expand the number of green reducing agents for the synthesis of 1D Ag nanostructures. Moreover, future studies are expected to focus on the effect of reaction parameters on yield and aspect ratio of 1D Ag nanostructures in these novel green and sustainable synthesis processes to not only tune the generation or deposition rate of silver atoms, but to control the surface diffusion rate of adatoms as well. Finally, due to the intrinsic instability of 1D Ag nanostructures because of their large surface to volume ratio, it is essential to manipulate the reaction conditions and parameters to preserve their 1D structure during the green synthesis process.

### 6 Current state and key challenges in the green synthesis of 1D Ag nanostructures

There are few green and sustainable methods of 1D synthesis of Ag nanostructures, where most of the syntheses are small-scale for a feasibility study. There are also inherent challenges in batch synthesis, such as challenges in batch-to-batch reproducibility. Moreover, there is a lack of fundamental understanding behind the synthesis reaction mechanism, and the functionality and responsibility of the specific compounds are not well understood. Without this fundamental knowledge, it is not possible to control and tune the size and morphology of the synthesized 1D Ag nanostructures, and there is a broad size distribution as well. By understanding the role of each functional group in the reduction, nucleation, and growth of silver nanostructures, it would be possible to predict the applicability of the other novel green reducing agents offered by nature for silver and consequently other 1D metal nanostructures synthesis.

#### 6.1 Inherent challenges in small-scale green synthesis of 1D silver nanostructures

A major gap in green 1D silver nanostructure synthesis processes resides in the small-scale of their production with low efficiency and predictability, which has not been implemented beyond the bench. The reasonable control of the synthesis process of green synthesized 1D silver nanostructures is still a challenge in their batch synthesis along with the challenges in batch-to-batch reproducibility. It is essential to investigate the effect of all of the reaction parameters on the size and morphology of the synthesized 1D Ag nanostructures, and find the most essential parameters that predominately control the morphology and yield of 1D Ag nanostructures. Moreover, it is important to investigate not only the simultaneous effect of these parameters but also to find the optimal reaction conditions at which the yield of 1D Ag nanostructures is maximized.

#### 6.2 Lack of recipe in the green synthesis of 1D silver nanostructures

Countless numbers of plant extracts from different parts of plants have been reported for the synthesis of silver nanoparticles. Although there are many published studies regarding the green synthesis of silver nanoparticles, there are comparatively few regarding 1D ones. Moreover, among these few studies, some of them are by the assistance of microwave or ultraviolet irradiation process and high energy requirements. Not only there are few studies, but all of them are in very small-scale reactions with low yield of 1D nanostructures compared to other nanostructures. This indicates a huge gap in the green synthesis of 1D Ag nanostructures and the necessity to expand the number of green reducing agents, specifically plant-based ones offered by nature for synthesis of 1D Ag nanostructures.

#### 6.3 Lack of fundamental understanding behind the green synthesis of 1D silver nanostructures

The concept of size, morphology, and shape control of silver nanostructures can be achieved by investigation of the growth mechanism and adjusting synthetic parameters. The advantages of utilizing different green reducing agents such as plants and plant-derived reagents for silver nanoparticle synthesis have been of interest in studies to evaluate the mechanism behind the synthesis process including uptake of the metal ions, their bioreduction, and the possible growth route for nanoparticle formation. However, there is a lack of fundamental knowledge regarding the reaction mechanism of green synthesis of 1D silver nanostructures, specifically environmentally friendly large-scale synthesis, a process with strong potential to overcome the challenges in chemical synthesis of 1D silver nanostructures. After finding the specific biochemical compound(s) or ingredient(s) in the green reducing agents that is responsible for the reduction of metal ions and consequent growth of nanoparticles to a desired morphology, it is essential to investigate the reaction mechanism, which would be crucial to control the reaction and consequently control the size, shape, and morphology of the synthesized 1D silver nanostructures. This would be achievable by understanding the exact route including reduction, nucleation, and growth of their green and sustainable synthesis process.

### 7 Lessons from green synthesis of silver nanoparticles towards green synthesis of 1D silver nanostructures

**Figure 2:**
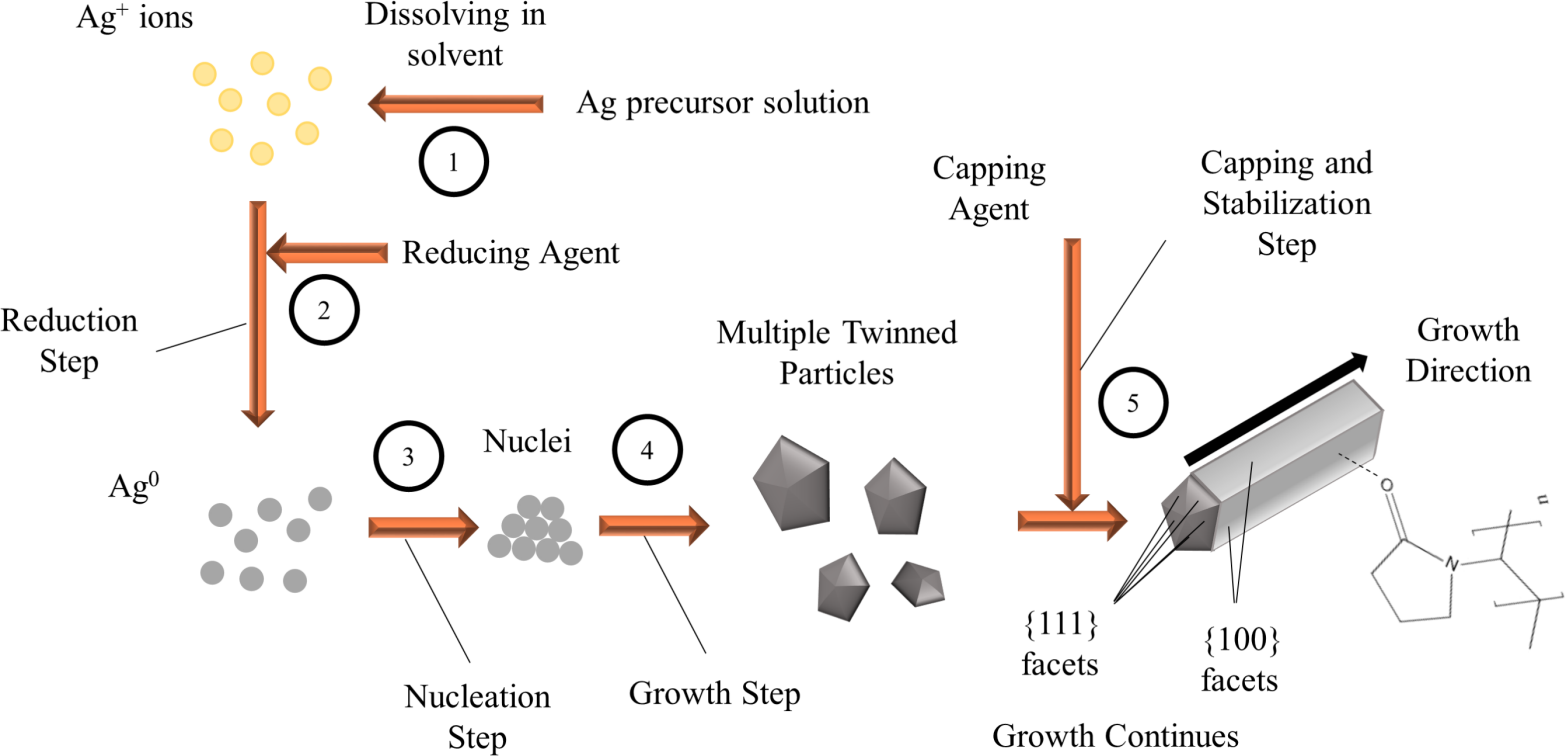
Synthesis steps and reaction mechanism for AgNW polyol synthesis.

Many green reagents can act as reducing and capping/stabilizing agent at the same time. However, this depends on the reaction mechanism and molecular structure of the reagent and its ingredients including particular functional groups such as amines, aldehydes, and carboxylates, to name a few [199]. Understanding the reaction mechanism is essential to tune and control the size and morphology-dependent properties of silver nanostructures. An important aspect in achieving 1D Ag nanostructure is identifying key factors controlling the stabilization and growth of initially formed Ag seeds. Nevertheless, a clear description of how natural organic molecules can direct the growth of AgNWs remains absent and only a few studies have discussed a possible mechanism in that regard. Fortunately, the mechanism of AgNWs synthesis via the polyol process is better understood and can be a useful guide in understanding the reduction process and diagnosis of responsible functional groups and binding mechanisms which direct the growth of 1D Ag nanostructures in a green synthesis process. In the case of polyol synthesis, PVP controls the capping/stabilizing mechanism, ethylene glycol controls the reduction process, and the temperature and the pH are among the factors that control the kinetics. In the polyol process, PVP binds to {100} facets of initially formed seeds through Ag–O bonding [199], and directs the growth through the {111} facet (Figure 2). In fact, the adsorption on {100} facets will be much stronger compared to {111} facets for the growth to take place [90]. In the study performed by Lin et al., polyhydroxy components such as alkaloids, flavonoids, and polysaccharides acted as capping ligands for the growth of AgNWs [370]. Many of the mentioned components contain carbonyl groups similar to PVP, which could possibly be responsible for ligand bonding. In addition, Lin et al. stated that the Ostwald ripening process was one of the factors in growth of AgNWs, which was also reported previously in the polyol process [381]. In another study performed by Dong et al., the tannin molecules bound to four {100} facets of AgNWs, leaving two other {100} facets free for growth [291]. The tannin molecule also has carbonyl groups, which might have been taking part in the formation of ligand bonds. In a recent work, Villalpando et al. used *Lavandula angustifolia* extract to synthesize AgNWs [382]. They mentioned that some compounds such as saponins and glycosidics that are present in the extract [383], are responsible for 1D growth of nanostructures. However, Ag–ligand interactions are part of a larger chain in the synthesis process and are related to other factors such as reduction rate, complex formation, and temperature. For instance, as mentioned earlier in the study conducted by Dong et al., higher temperatures (>50 °C) made the conditions difficult for tannin molecules to effectively bind themselves (due to lack of adequate time) to {100} facets of AgNWs, in which nanoparticles were formed instead of nanowires [291]. In addition, tannin molecules are able to form stable complexes with Ag^+^ ions [369], which results in decreasing the reduction rate, paving the way for 1D anisotropic growth [384]. It was also previously mentioned that acidic pH is more in favor of AgNW formation due to moderation of the reduction rate [291,375]. For instance, Dong et al. showed that the yield of AgNWs decreased by increasing the pH up to 9, while the yield increased by lowering the pH to 5 [291]. However, further decrease resulted in a smaller yield, which was attributed to an excessive decrease in the reduction rate. Nadagouda et al. synthesized AgNRs in the presence of vitamin B2 in aqueous solution and reported that the self-assembly of AgNRs was due to strong interparticle van-der-Waals and hydrophobic interactions [204]. The use of exotic seeds was therefore not needed due to the self-assembly. In addition, vitamin B2 was able to form complexes with Ag^+^ ions and help to reduce the reaction rate, which leads to anisotropic growth of AgNRs. Wang et al. synthesized AgNWs using glucose in hydrothermal conditions (180 °C and under autoclave), and reported that the lower the concentration of Ag^+^, the more favorable the anisotropic growth of AgNWs. They added NaCl to the AgNO_3_ solution in order to form AgCl colloids. Due to the low solubility of AgCl in water, the concentration of Ag^+^ is low at 180 °C, which was favorable to growth of AgNWs. On the contrary, AgNPs were formed when only AgNO_3_ was used. In the latter condition, the reduction rate of Ag^+^ ions is higher, which does not allow growth of AgNWs. Another important factor is the diffusion of initially formed seeds. Villalpando et al. recently reported that centrifugation is able to enhance the diffusion rate and therefore favor the growth of AgNWs [382]. They reported that AgNW growth is affected by coalescence and interparticle diffusion mechanism as a result of a secondary nucleation process, where smaller particles are coalesced into forming AgNWs, as described previously [385]. The enhancement of diffusion and growth of AgNWs using magnetic stirring was also reported previously [386].

## Conclusion

The synthesis process is important to determine the size and morphology of Ag nanostructures. The green procedures for the synthesis of silver nanostructures in addition to the most common chemical and physical methods were reviewed. The advantages of green synthesis methodologies, such as their low cost, low energy consumption, scale-up capability, and simplicity that outweighs their disadvantages compared to the chemical and physical synthesis methodologies were discussed. Green synthesis processes were classified among which synthesis of Ag nanostructures using plant extracts are considered to be a promising route due to their simple utilization, aqueous nature, and non-toxic properties. Plant extracts are likely to contain certain natural compounds such as polyphenols, flavonoids, alkaloids, and different functional groups such as hydroxy groups and carboxylic acids, which can provide strong reducing and capping capability. Due to their broad scope, plant extracts are used for preparation of various Ag nanostructures with different sizes, and the literature studies on this subject have been growing during the last decade. The different green reagents based on studies in the literature at different reaction conditions to synthesize Ag nanostructures with different sizes and morphologies were outlined. In addition to reagent type, the synthesis of Ag nanostructures using plant extracts is highly dependent on various process factors such as temperature, reagent concentration, and pH that were discussed. The green synthesis of 1D Ag nanostructures using plant extracts is gaining more attention and various compounds such as tannins, glucose, clove oil, and other natural extracts may be used as both reducing and capping agents to synthesize 1D Ag nanostructures. Unlike the polyol process, the green route consumes much less energy and is simpler. The aspect ratio of 1D Ag nanostructures is dependent on the temperature, pH, solvent, and reagent type. The 1D Ag nanostructure formation mechanism, and how those factors can be tailored to increase their yield were discussed. The advantages of the green synthesis techniques make it a valid and promising platform for facile and high-yield synthesis of 1D Ag nanostructures such as AgNRs and AgNWs.

**Figure 3:**
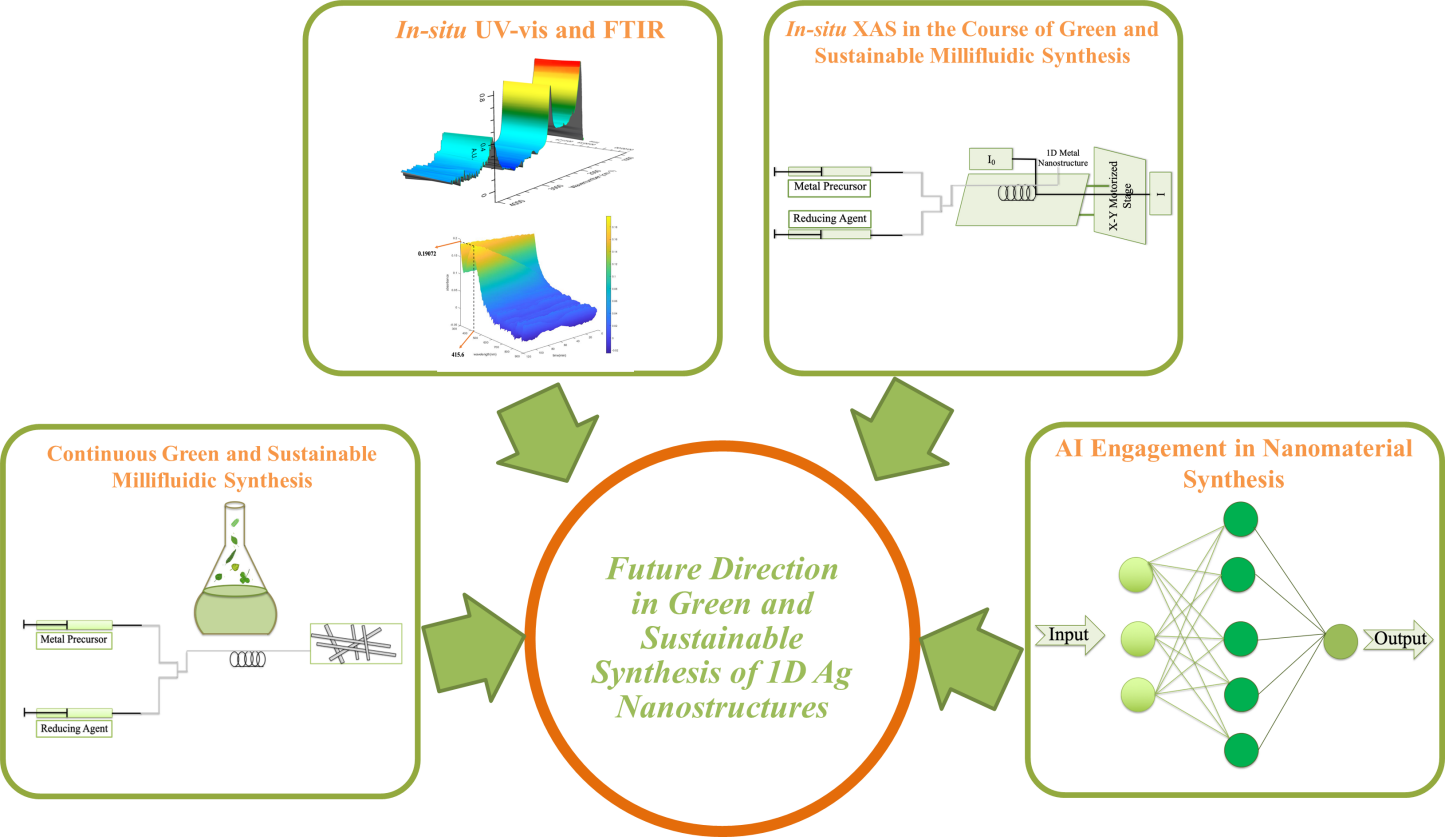
Expected future direction in green and sustainable synthesis of 1D Ag nanostructures.

The current advances in in situ quantitative understanding using different instrumentation in the course of the reaction, including reduction, nucleation, and growth, will allow precise control of the size and morphology of metal nanostructures. In situ UV–vis can be used not only to investigate the optical properties of metal nanoparticles, but to monitor their quantitative formation and size as well. In situ FTIR spectroscopy can be used to find the different functional groups responsible for the reduction of metal ions and stabilization of metal nanoparticles from the peak positions in the spectrum. Future research is expected to focus on integration of these in situ characterization techniques to the batch green synthesis of 1D silver nanostructures to come up with the exact reaction mechanism, and consequently not only to control their size and morphology, but to expand the number of reducing and capping agents offered by nature for the synthesis of 1D silver nanostructures as well. Moreover, future research is expected to focus on large-scale industry relevant 1D silver nanostructure production in a green, sustainable, and continuous manner. The design of novel laboratory flow reactors has the potential to reduce waste, minimize the building space and energy requirements, and yield more accurate predictive models during development and manufacturing. The flow reactors, such as microfluidic reactors, offer uniform heat and mass transfer, more homogenous mixing of reagents, higher yield, and throughput, and are adaptable for in situ monitoring characterizations. However, the fabrication of microfluidic devices is time- and resource-intensive, and requires complicated facilities that support the advantages of millifluidic flow reactors in nanoparticle synthesis. These novel reactor systems offer similar advantages to microfluidic ones, while being easier to fabricate, simpler to reconstruct, and even more adaptable for in situ monitoring characterizations. A larger surface to volume ratio and precisely controlled flow patterns that consequently increase heat and mass transfer rate, coupled with inherent safety are among other advantages of flow millifluidic platforms [66,387–388]. The application of a continuous millifluidic reactor has the potential to be a method to overcome the challenges in batch green 1D silver nanostructure synthesis through control of a uniform chemical and thermal reaction environment in a small reaction volume. Novel in situ characterization techniques are essential for reaction mechanism investigation to control the morphology of the synthesized 1D Ag nanostructures in a continuous millifluidic reaction as well. In situ X-ray absorption spectroscopy (XAS) is a versatile technique that provides an opportunity to investigate the reaction dynamic and mechanism of 1D metal nanostructure growth in millifluidic reactor to further control their morphology, size distribution, and crystal structures. This expected future research direction will enable a synthesis route where 1D Ag nanostructure properties can be selected and tuned by simple alteration of reaction parameters and millifluidic reactor design to produce these nanostructures in an industrial relative scale in a green and sustainable manner. Furthermore, it is critical to optimize these green and sustainable techniques, not only in terms of scale-up capability, but also with respect to product quality and performance. Finally, the application of artificial neural network (ANN) tools in nanomaterial synthesis such as 1D silver nanostructures will save time and expenditure by predicting the reaction outcomes in which the most desirable nanomaterial size, morphology, and yield is achieved. Data from different characterization techniques including scanning electron microscopy (SEM), transmission electron microscopy (TEM), UV–visible spectroscopy (UV–vis), Fourier-transfer infrared spectroscopy (FTIR), and X-ray absorption spectroscopy (XAS) may be utilized to train the network to optimize the green synthesis of silver nanostructures as well as to predict the size and morphology of the synthesized silver nanostructures at different reaction conditions. It will consequently have a significant impact on industrial large-scale manufacturing of silver nanostructures, in which new molecule discovery as well as the development of alternatives to existing chemical processes will be discovered. Ultimately it will address the principal need in coupling green metal nanostructure synthesis, novel in situ characterization techniques, and artificial intelligence (AI) tools. A schematic on future direction in green and sustainable synthesis of Ag nanostructures with focus on 1D ones is shown in Figure 3.
